# Competition between commensal protists shapes gut mucosal immunity in mice

**DOI:** 10.1128/mbio.00802-26

**Published:** 2026-05-18

**Authors:** Marienela Y. Heredia, Paul A. Kuehnert, Kylie March, Laura J. Knoll, Claire E. O'Leary

**Affiliations:** 1 Department of Medical Microbiology and Immunology, University of Wisconsin—Madison School of Medicine and Public Health 732057, Madison, Wisconsin, USA; 2 Department of Pediatrics, University of Wisconsin—Madison School of Medicine and Public Health 200763, Madison, Wisconsin, USA; Georgia Institute of Technology, Atlanta, Georgia, USA

**Keywords:** *Entamoeba*, *Tritrichomonas*, tuft cell, type 2 immunity

## Abstract

Intestinal protists are emerging as key modulators of host immunity and microbial ecology, yet their impact on mammalian hosts remains poorly defined. Here, we investigated the role of two distinct protists, the amoeba *Entamoeba muris*, and the parabasalid, *Tritrichomonas*, to determine how they shape gut immunity *in vivo* individually and together. Unlike the well-characterized inducer of type 2 immunity, *Tritrichomonas*, which activates the tuft cell–IL-25–ILC2 circuit in the small intestine, *E. muris* failed to elicit robust immune responses in the intestine or colon. However, the introduction of *E. muris* into mice naturally colonized by *Tritrichomonas* spp., or co-infection with *E. muris* and *Tritrichomonas* spp. reduced *Tritrichomonas*-induced type-2 response in the small intestine and *Tritrichomonas-*dependent immune activation in the colon. Our data suggest that *E. muris* may limit the abundance of *Tritrichomonas* spp., with reduced protist loads in the cecum specifically correlating with diminished tuft cell activation. We also identified sex-specific differences in the intestinal response to *Tritrichomonas* spp., which have not previously been reported. Taken together, these findings reveal that the presence of *E. muris* reduces *Tritrichomonas*-dependent activation of type 2 immunity in the small intestine, even when both protists can be detected in the cecum, without triggering overt inflammation. This work provides a framework for understanding how protists interact within the gut ecosystem and shape mucosal immunity in the absence of pathogenicity.

**IMPORTANCE:**

Single-cell parasites called protists are common in mammalian intestinal tracts, yet their modulation of the host immune response and interactions with each other remain poorly defined. Here, we investigated the role of two protists, *Entamoeba* and *Tritrichomonas*, to determine how they shape gut immunity individually and together. Unlike the well-characterized inducer of type 2 immunity, *Tritrichomonas*, which activates the tuft cell circuit, *Entamoeba* failed to elicit a robust immune response. The introduction of *Entamoeba* into mice naturally colonized by *Tritrichomonas*, or co-infection with Entamoeba and Tritrichomonas, reduced the *Tritrichomonas*-induced immune response. Our data suggest that *Entamoeba* limits the abundance of *Tritrichomonas*, correlating with diminished tuft cell activation. We also identified sex-specific differences in the intestinal response to *Tritrichomonas*. These findings show that *Entamoeba* reduces *Tritrichomonas*-dependent activation of type 2 immunity without triggering much inflammation. It helps our understanding of how protists interact within the gut and shape immunity without disease.

## INTRODUCTION

Many protists establish long-lasting residence in the mammalian gastrointestinal tract (amoebae, parabasalids, apicomplexans, diplomonads, ciliates, and dinoflagellates), but protists remain understudied members of the gut microbiome (1–3). Protists can remodel the host bacterial microbiota and the intestinal immune landscape through direct and indirect pathways (1, 4, 5), and, when present, may be keystone species within the host microbiome.

The protist genus *Entamoeba* is best known for the disease-causing parasite, *Entamoeba histolytica*, which can cause invasive amebiasis (6). Although the pathogenesis of *E. histolytica* invasive disease is well understood, little is known about non-pathogenic *Entamoeba* infections*,* even though most infections are asymptomatic (7). Non-pathogenic *Entamoeba* spp. can alter the bacterial microbiota (8), but explorations of *Entamoeba* interactions with the host immune system have been limited. Recently, the mouse commensal amoeba, *Entamoeba muris*, was identified as a model organism for studying fecal-oral transmission of *Entamoeba* spp. *E. muris* is phylogenetically related to the human-colonizing, non-pathogenic *Entamoeba coli* (9, 10). Significant changes to the bacterial microbiome have been observed in both BALB/c and C57BL/6J background mice infected with *E. muris* (11, 12)*. E. muris*-infected C57BL/6J mice were protected against high-fat diet-induced metabolic syndrome and steatotic liver disease, with infected mice demonstrating reduced inflammation (12). In contrast, BALB/c mice infected with *E. muris* developed colonic inflammation with eosinophilia and T-cell infiltration (12). These studies suggest that immunological consequences of *E. muris* infection may be context dependent.

Local and systemic immune effects are now well characterized in mice colonized with parabasalids, specifically murine tritrichomonads. In the distal small intestine, succinate is released by *Tritrichomonas* spp. initiates a type-2 immune response driven by activation of specialized epithelial cells called tuft cells, which express the succinate receptor, SUCNR1 (13–16). Activated tuft cells release interleukin (IL)−25, which activates group 2 innate lymphoid cells (ILC2s) to produce IL-5 and IL-13; IL-13, in turn, promotes increased tuft and goblet cell differentiation (13, 17). Sustained activation of this tuft cell-IL-25-ILC2 “circuit” results in tuft and goblet cell hyperplasia, smooth muscle hyperplasia, intestinal lengthening, and activation of negative regulatory mechanisms (15, 18–20). In the colon, *Tritrichomonas* spp. impact several mucosal immune populations, promoting expansion of Th1 and Th17 cells (21–23); these effects do not require tuft cells. Tritrichomonads also exert systemic effects on host immunity through induction of non-protist-specific IgA and tuft cell-dependent and -independent effects on the host bacterial microbiome (16, 24–29).

While recent work has explored the interaction between two metabolically distinct *Tritrichomonas* species (*T. musculus*, previously identified as *T. muris/T. musculis* (3), and *T. casperi*) (16), and others have also identified multiple species of *Tritrichomonas* within a single murine host (23), no study to date has explored the mucosal immune response in the presence of protists from distinct phyla. This is despite the tremendous diversity of protist phyla found in the human gastrointestinal tract, in which two or more distinct phyla can be observed (30).

Here, we investigated how non-invasive *Entamoeba* colonization influences the gut mucosal immune landscape using *E. muris* infection in C57BL/6J mice, and how two distinct protist phyla shape gut mucosal immunity using two models of *E. muris* and *Tritrichomonas* spp. co-infection. We found that *E. muris* has minimal impact on mucosal immune cells along the gastrointestinal tract in C57BL/6J mice. However, infection by *E. muris* leads to decreased activation of the tuft cell-IL-25-ILC2 circuit in mice co-infected with *E. muris* and *Tritrichomonas* spp., ultimately influencing the mucosal immune response in the small intestine as well as the colon. These data suggest not only that distinct protists affect mammalian gut immune responses in unique ways but also that interactions among protist families, orders, or phyla can dramatically impact the capacity of a single species or genus to exert immunomodulatory effects, implying that identifying the presence of a single protist is insufficient to make conclusions about the immunological consequences of infection.

## RESULTS

### C57BL/6J mice naturally colonized by *E. muris* exhibit cyclic shedding

**Fig 1:**
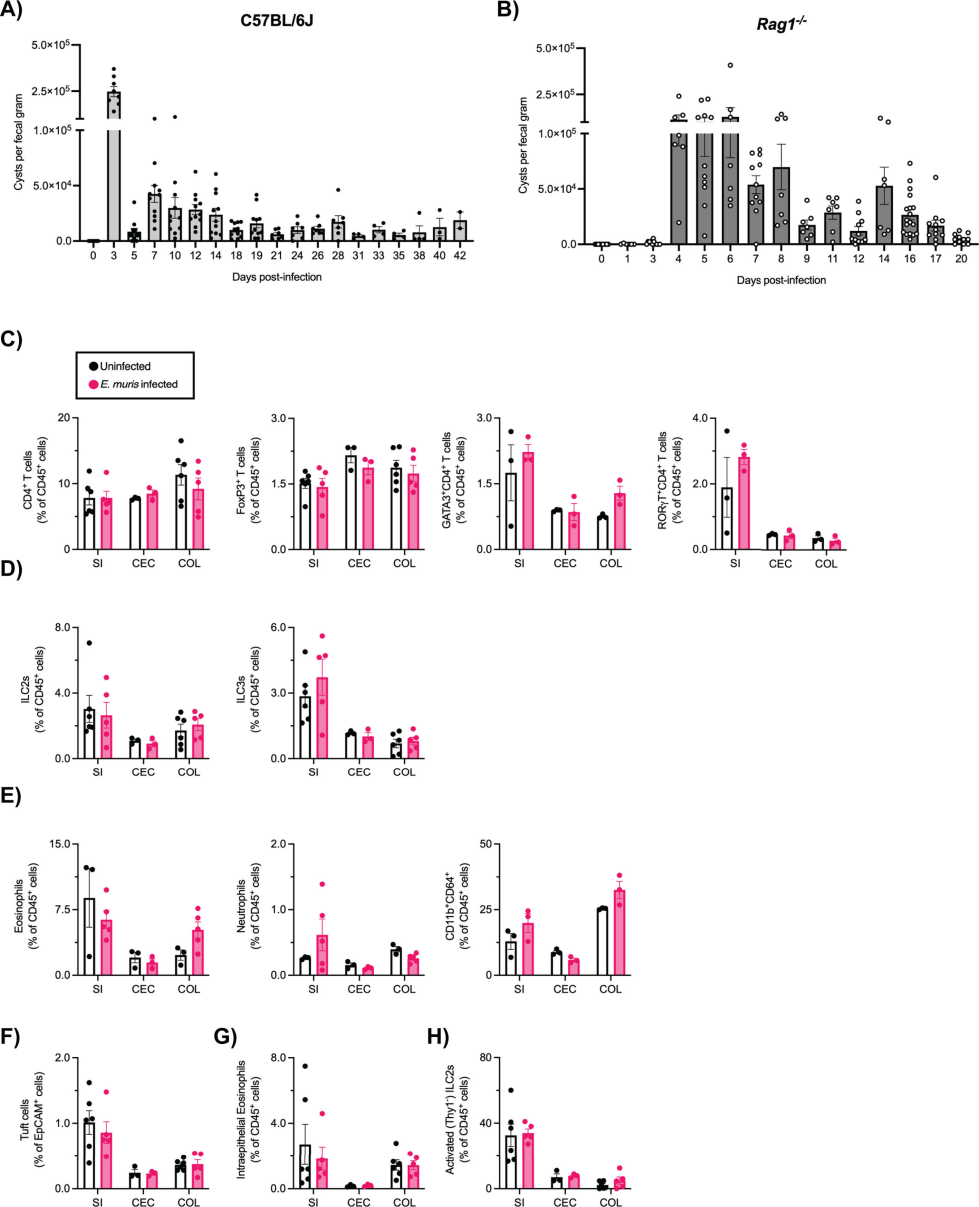
C57BL/6J mice naturally colonized by *E. muris* exhibit cyclic shedding but no significant changes in intestinal immune cells. (**A**) Fecal cyst counts post *E. muris* infection in C57BL/6J and (**B**) *Rag1^−/−^* mice. Counts were normalized to the mass of fecal starting material. (**C–E**) Analysis of immune cell populations by flow cytometry within the lamina propria fraction of distal small intestine (SI), cecum (CEC), and colon (COL) tissue (28 dpi) as a frequency of CD45+. (**F and G**) Analysis of tuft cell (frequency of EpCAM+) and intraepithelial eosinophil (frequency of CD45^+^) populations within the epithelial fraction of SI, CEC, and COL tissue 28 dpi. (**C**) Total CD4+ T cells (left), including FoxP3+ (left-middle), GATA3+ (right-middle), and RORγT+ (right) subsets. (**D**) ILC2s (Lin-Thy1+GATA3+) and ILC3s (Lin-Thy1+ RORγT+). (**E**) Eosinophils (SiglecF+CD11b+Ly6G+CD64+), neutrophils (Ly6G+CD11b+CD64−), and CD64+CD11b+SiglecF−Ly6G− immune cells. (**F**) Tuft cells (CD24+IL−25−RFP+), (**G**) intraepithelial eosinophils (SiglecF+CD11b+Ly6G−CD64−), and (**H**) activated ILC2s (Thy1−) as a frequency of CD45 + cells, respectively, from the epithelial fraction of the same tissues analyzed by flow cytometry. (**C–H**) Error bars represent the standard error of the mean (SEM). Significance was determined by one-way analysis of variance (ANOVA), and all non-significant comparisons were omitted from panels C−H for visual clarity. Flow cytometry data are representative of two independent experiments with a total of six controls and five *E. muris*-infected mice.

Previously, we developed a fecal-oral transmission model of *Entamoeba* infection in Swiss-Webster (SW) mice using infectious cysts isolated from fecal matter of C57BL/6J mice naturally colonized by *E. muris* (10). At 28 days post-infection (dpi)*,* cyst shedding in SW mice is negligible, but mice remain colonized in the hind gut long-term. The kinetics of infectious cyst shedding in the C57BL/6J background after primary infection have not been described. Therefore, we orally infected *E. muris*-negative C57BL/6J mice and tracked fecal cyst shedding via sucrose gradient counts. We observed continuous shedding of viable, infectious cysts (Fig. 1A; Fig. S1A) up to 28 dpi. As these C57BL/6J background mice were littermated *Ripk3^−/−^* (necroptosis-deficient [31–33]) and wild-type controls, we assessed whether shedding was impacted by loss of *Ripk3*. We observed no difference in fecal cyst shedding between wild-type mice and *Ripk3*^−/−^ mice (Fig. S1B). Additionally, cyst shedding was not sex dependent (Fig. S1C).

During infection, other protists display fluctuations in host burden/shedding due to antigenic variation, an adaptive immune evasion strategy (34). Given the chronicity of *E. muris* infection in our C57BL/6J mice and fluctuations in cyst shedding abundance, we hypothesized that pressure from the adaptive immune system could drive *E. muris* encystation. To test this, we orally infected B and T cell-deficient *Rag1*^−/−^ mice (35) with *E. muris. Rag1*^−/−^ mice in the C57BL/6J background also demonstrated persistent and fluctuating cyst shedding, suggesting that adaptive immune cells do not influence cyst shedding dynamics (Fig. 1B; Fig. S1D). This contrasts with the lack of fecal shedding recently reported in immunodeficient and T cell-depleted BALB/c mice (11).

### *E. muris* has minimal impact on immune responses in the gut

In humans, most *Entamoeba* infections, including *E. histolytica*, never result in immunopathology or symptomatic disease (1, 5–8). To define the immune responses to *E. muris* in C57BL/6J mice, we analyzed major immune cell populations by flow cytometry analysis of lamina propria cells from the distal small intestine, cecum, and colon of uninfected and *E. muris*-infected mice 28 dpi. *E. muris* infection was confirmed via qPCR (Fig. S1E). We observed no significant differences in major populations of T cells (Fig. 1C), innate lymphoid cells (Fig. 1D), or myeloid populations (Fig. 1E; see gating strategy, Fig. S1F through H), in contrast to recent findings in BALB/c mice (11).

Given the association between protist colonization and ILC2/tuft cell activation established with *Tritrichomonas* spp. (36) and recently reported for *Giardia* (37), we examined this cellular axis using IL-25 reporter mice (“Flare25”) (17). We observed no changes in IL-25^+^CD24^+^ tuft cells or intraepithelial eosinophil frequencies, a proxy for IL-5 levels, in *E. muris*-infected mice (Fig. 1F and G). Using loss of Thy1 as a marker for ILC2 activation (38), we also did not observe any change in the frequency of activated ILC2s in lamina propria between *E. muris*-infected mice (Fig. 1H). Taken together, these data suggest that *E. muris* does not significantly alter the frequencies of major gut immune cell populations in C57BL/6J mice.

### *Tritrichomonas* spp.-mediated activation of the tuft cell-IL-25-ILC2 circuit is lost upon *E. muris* infection

**Fig 2:**
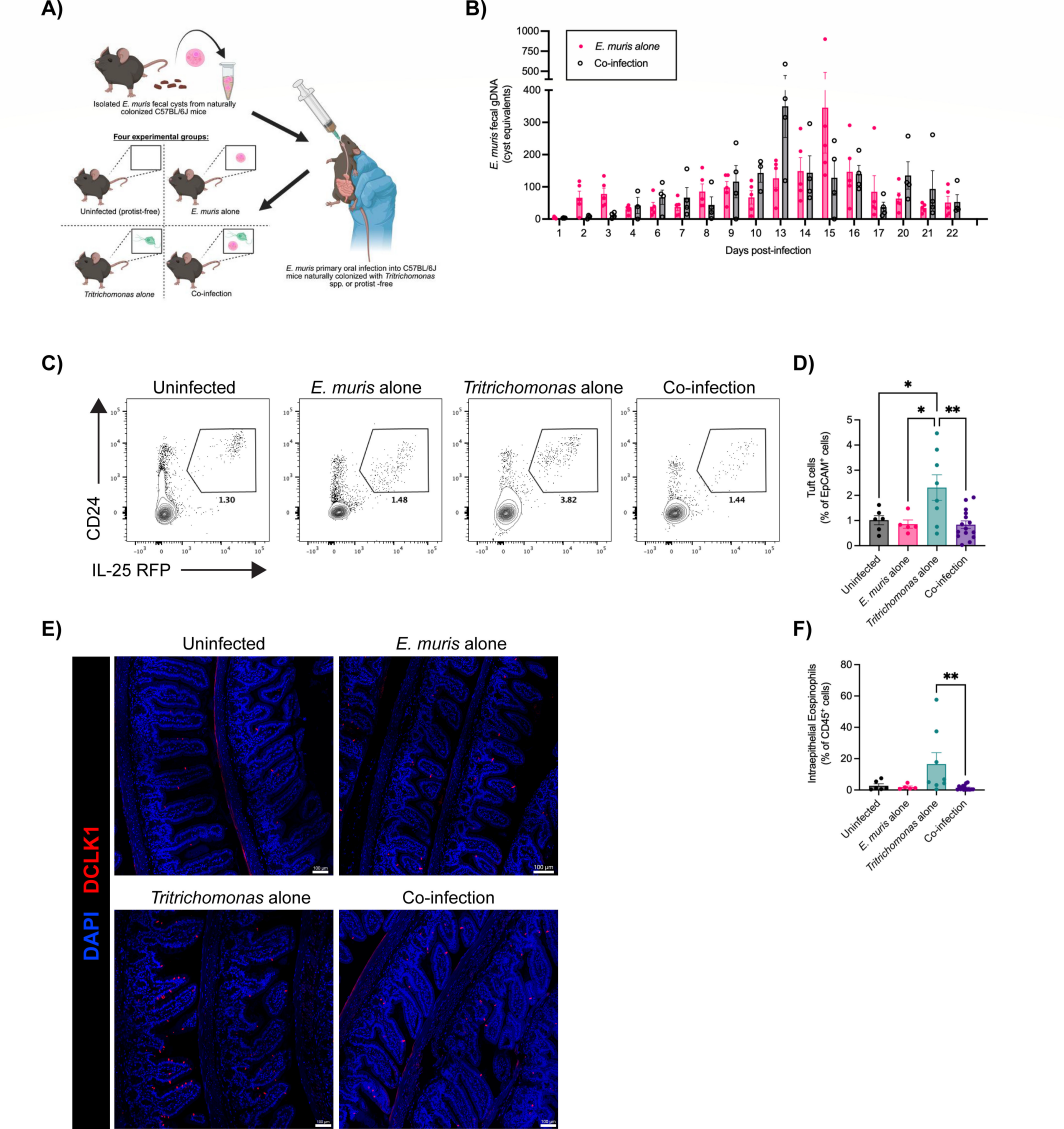
Loss of intestinal tuft cell-IL-25-ILC2 circuit activity in *Tritrichomonas* spp.-colonized mice infected with *E. muris*. (**A**) Study design of naturally colonized *Tritrichomonas* and protist-free (PCR negative for *E. muris* and *Tritrichomonas* DNA) in-house bred infected with *E. muris* cysts by oral gavage. Terminal analysis was conducted on mice 28 dpi. (**B**) *E. muris* primers were used for qPCR analysis of fecal genomic DNA (gDNA), quantified against a standard curve of DNA isolated from purified *E. muris* cysts (“cyst equivalent”). (**C and D**) Tuft cells were analyzed using flow cytometry (CD24+IL25^RFP/RFP^+) and quantified as a frequency of epithelial cells (EpCAM+) from the distal small intestine. (**E**) Representative images of distal small intestine Swiss rolls stained with an antibody against DCLK1 (red) to identify tuft cells; counterstained with 4′,6-diamidino-2-phenylindole dihydrochloride (DAPI; blue). (**F**) Ileal intraepithelial eosinophils as a frequency of CD45+ cells. (**D and F**) *P* values calculated by ordinary one-way analysis of variance followed by Tukey’s multiple comparisons test *P < 0.05 and **P < 0.005.

The impact of succinate-secreting species of *Tritrichomonas* on host mucosal and systemic immunity and the gut microbiome is clear, but the interaction between *Tritrichomonas* and other protist endobionts has not been examined. To address this, we orally infected in-house bred C57BL/6J “protist-free” mice (meaning PCR negative for fecal *E. muris* and *Tritrichomonas* DNA) and C57BL/6J mice naturally colonized with *Tritrichomonas* spp. with *E. muris* cysts (Fig. 2A). In both co-infected mice and mice infected with *E. muris* alone, we observed persistent fecal shedding of *E. muris* (Fig. 2B; Fig. S2A and B). The level of *E. muris* fecal DNA determined by qPCR was not significantly different between coinfected mice and mice infected with *E. muris* alone. Mice in which there was no detectable *E. muris* DNA in fecal pellets following initial infection were considered failed infections and excluded from all analyses (Fig. S2C).

To test the impact of co-infection on *Tritrichomonas*-mediated activation of the tuft cell-ILC2 circuit, we performed flow cytometry analysis of distal small intestine epithelial cells. The frequency of tuft cells was significantly reduced in co-infected mice compared to mice harboring *Tritrichomonas* spp. alone (Fig. 2C and D). This was corroborated by immunofluorescence imaging (Fig. 2E). Consistent with this, the ILC2 frequency in small intestine lamina propria of co-infected mice was reduced compared to mice colonized with *Tritrichomonas* spp. (Fig. S2D). ILC2s from *Tritrichomonas* spp.-colonized mice rarely expressed Thy1, suggesting that the majority are in an activated state; this was normalized in co-infected mice (Fig. S2E). Increased cell death under conditions of elevated type 2 immunity (15, 38) limited extensive analysis of ILC2 activation/frequency in the distal small intestine. However, intraepithelial eosinophil frequencies were significantly reduced in co-infected mice compared to *Tritrichomonas spp*. colonized mice (Fig. 2F), consistent with reduced type 2 cytokines. Taken together, these data suggest that 4 weeks following co-infection with *E. muris*, the stereotypical type 2 immune response induced by *Tritrichomonas* is significantly blunted.

### *Tritrichomonas* burden in naturally colonized hosts is reduced upon *E. muris* infection

**Fig 3:**
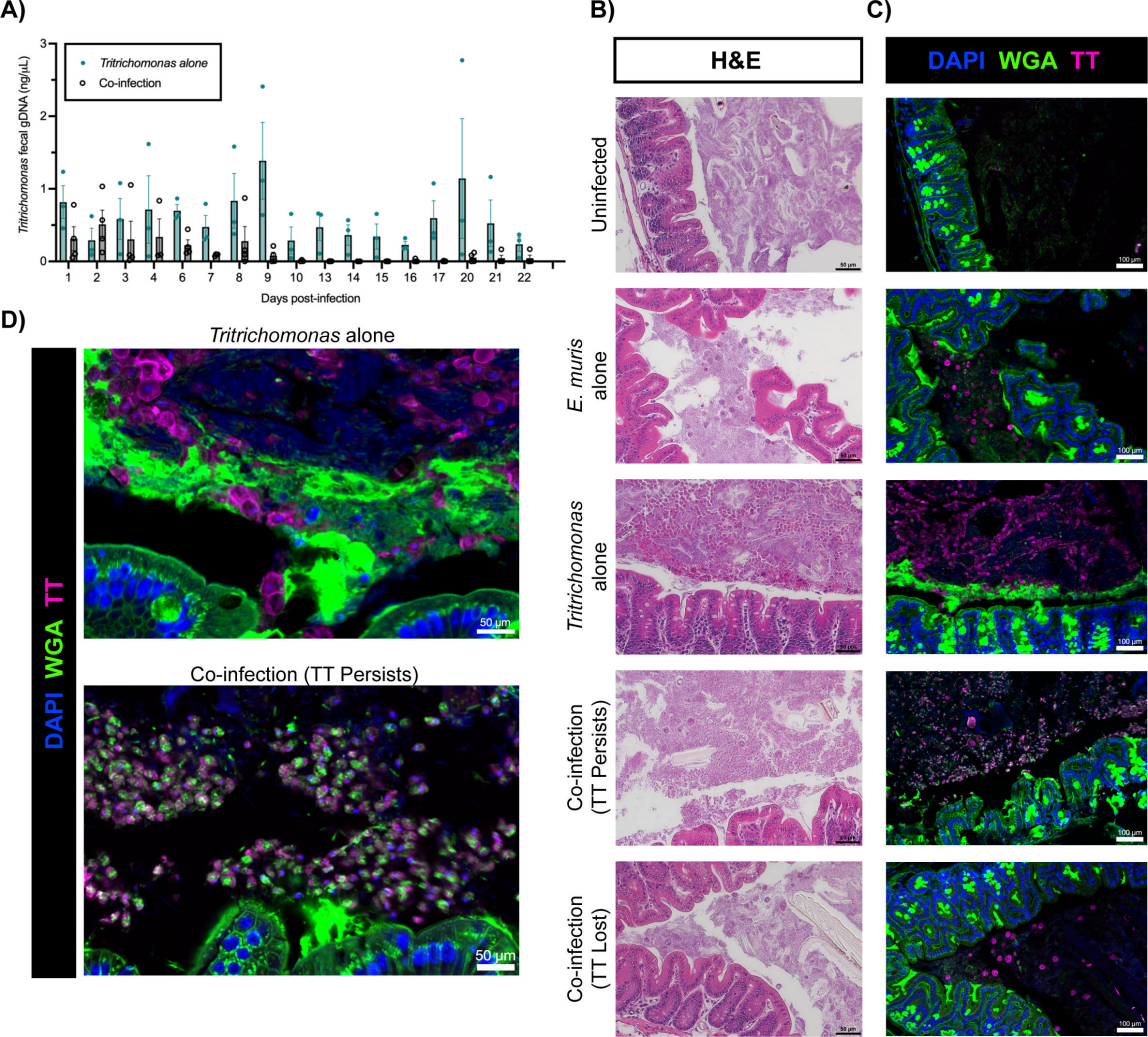
*E. muris* infection leads to reduced *Tritrichomonas* burden in naturally colonized hosts. (**A–D**) Naturally colonized *Tritrichomonas* and control mice were infected with *E. muris* as described in Fig. 2A. (**A**) *Tritrichomonas* burden was quantified by qPCR from gDNA extracted from fecal pellets. Infection status was a significant factor by two-way analysis of variance using a mixed effects model for missing values: *P* = 0.0032. (**B**) Hematoxylin and eosin (H&E) stained cecal sections suggest the presence of trophozoites of varying size relative to uninfected controls. (**C**) Representative images of ceca stained with antibody against *Tritrichomonas* (TT, pink) and wheat germ agglutinin (WGA, green), counterstained with 4′,6-diamidino-2-phenylindole dihydrochloride (DAPI; blue). (**D**) High magnification images (40×) of *Tritrichomonas* alone and co-infection (TT persists) ceca from panel C.

We hypothesized that in co-infection *Tritrichomonas* levels might be reduced, leading to loss of tuft cell-ILC2 circuit activation. To test this, we quantified *Tritrichomonas* genomic DNA (gDNA) from fecal pellets. *Tritrichomonas* gDNA declined to near undetectable levels by 10 dpi in co-infected animals (Fig. 3A; Fig. S3A), suggesting that introduction of *E. muris* could reduce or eliminate *Tritrichomonas* burden. In some animals, however, *Tritrichomonas* DNA was still present at low levels.

While the loss of *Tritrichomonas* would explain the reduced activity of the tuft cell-ILC2 circuit, previous studies have demonstrated that distinct *Tritrichomonas* spp. differentially secrete succinate and do not always activate tuft cells. *T. musculus* primarily consumes dietary carbohydrates, secreting succinate to activate the intestinal tuft cell-ILC2 circuit, while *T. casperi* associates with the mucus barrier and primarily consumes mucus glycans without secreting succinate. When dietary microbiota-accessible carbohydrates are scarce, *T. musculus* can undergo a metabolic switch, relying on mucus glycans to sustain a smaller population of succinate-producing protists, leading to reduced tuft cell-ILC2 circuit activity despite the continued presence of *T. musculus* (16). Such mucus-associated microbes are often poorly captured by fecal analysis (39).

To determine whether pressure from *E. muris* infection led to a change in the dominant species of *Tritrichomonas*, we first assessed whether the previously described species could be identified in our in-house colonized mice. PCR analysis of cecal DNA with primers specific for *T. casperi* and *T. musculus* suggested the presence of both species, or related variants (Fig. S3B). Therefore, we sought to quantify the relative abundance of these species using qPCR on fecal and cecal content of co-infected mice. In the absence of monocultured protists, we attempted to use purified 18s amplicons as a synthetic standard for species-specific qPCR without success (Fig. S3C), limiting our ability to determine the impact of *E. muris* on specific species abundance.

As an alternative approach to determine whether co-infection impacted the relative abundance of these two species, which are notably different in size, or induced a metabolic switch in *T. musculus* driving mucosal localization, we assessed the presence and localization of protists in the cecum via hematoxylin and eosin (H&E) and immunofluorescence imaging with a recently described antibody raised against *T. musculus*, which is known to cross-react with *T. casperi* (anti-TT) (16) (Fig. 3B and C). *Tritrichomonas*-positive mice were robustly colonized, while *Tritrichomonas-*negative control mice were devoid of cecal protists. Cecal H&E staining of *E. muris*-infected mice revealed the presence of round, amoeboid structures consistent with the morphology of *E. muris* described by Kobayashi et al. (9). Similar round structures were obvious with the anti-TT antibody staining as well, suggesting cross-reactivity of this antibody across protist phyla.

While cross-reactivity of the anti-TT antibody with *E. muris* limits its utility for definitive protist identification during co-infection, morphologically distinct structures were evident in both H&E and anti-TT-stained cecum from coinfected mice in which qPCR suggested persistent *Tritrichomonas* (TT persists) vs total elimination (TT lost; Fig. 3B through D; Fig. S3D). In these animals, we observed both large amoeboid structures, suggestive of *E. muris* cysts or trophozoites, alongside smaller protists which also co-stained with wheat germ agglutinin (WGA) and appeared more closely opposed to the cecal epithelium. Protists in mice naturally colonized with *Tritrichomonas* did not stain with WGA and were more commonly found in the tissue lumen and outer mucus layer or mucus plumes. While the small size and localization could be consistent with metabolic switching or *T. casperi*, WGA binding was recently described as a potential marker of *Tritrichomonas* pseudocysts (26). To rule out that these smaller protists juxtaposed to the epithelium were extravasating immune cells, which may have consumed protist antigen, we stained serial sections of the cecum with anti-CD45 antibody. No obvious immune cell infiltration was observed (Fig. S3E). These imaging data provide evidence for persistent *Tritrichomonas* following infection with *E. muris*, but whether this persistent *Tritrichomonas* is primarily pseudocysts or metabolically incapable of secreting succinate and activating tuft cells remains unknown.

### *E. muris* outcompetes *Tritrichomonas* spp. in a simultaneous co-infection model

**Fig 4:**
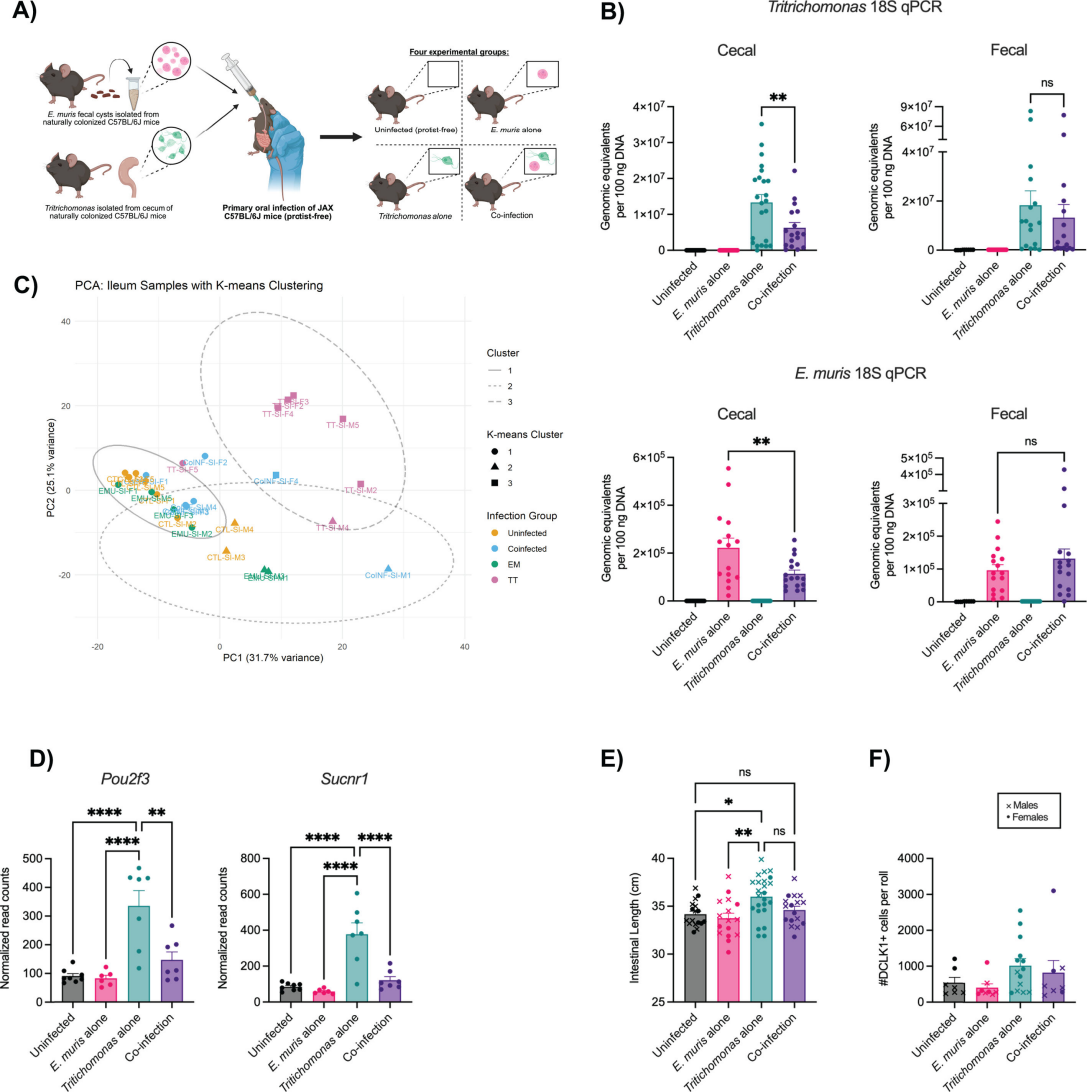
Simultaneous co-infection inhibits *Tritrichomonas*-mediated activation of the tuft cell-ILC2 circuit. (**A**) Study design of co-infection of *Tritrichomonas* and *E. muris* into protist-free C57BL/6J from Jackson Laboratories (“JAX” mice). All analyses were conducted at 28 dpi. (**B**) *Tritrichomonas* and *E. muris* burden was quantified by 18S qPCR analysis on extracted gDNA from cecal content (left) and fecal pellets (right). gDNA content was quantified against a standard curve against known genome copy number from a gel-purified amplicon specific to *Tritrichomonas* or *E. muris*. (**C**) Principal component analysis (PCA) and (D) expression of *Pou2f3* and *Sucnr1* by bulk RNA sequencing of the distal ileum. (**E**) Small intestinal length. (**F**) Tuft cell quantification based on antibody staining for DCLK1 in the entire section from an 8 cm distal intestinal Swiss roll. (**B and D–F**) Error bars represent the standard error of the mean (SEM). Ordinary one-way analysis of variance followed by Tukey’s multiple comparisons. ns, not significant; *P < 0.05, **P < 0.005, and ****P < 0.0001.

To further address the reduction of *Tritrichomonas* spp. in the presence of *E. muris*, we performed primary, simultaneous infection of *E. muris* and *Tritrichomonas* spp. into mice sourced from Jackson Laboratories (room EM09, “JAX mice”; Fig. 4A). We then analyzed the presence of *E. muris* and *Tritrichomonas* DNA in fecal pellets and cecal content. To better account for genome copy/ploidy variation (cyst vs trophozoite), we used purified *E. muris* or *Tritrichomonas* spp. amplicons to generate a standard curve of qPCR CT value vs genome copy number. In co-infected mice, we observed a significant decrease in genome copy number of *Tritrichomonas* spp. in cecal content but no reduction in fecal pellet copy number compared to *Tritrichomonas* mono-infected mice (Fig. 4B). This contrasted with the near elimination of *Tritrichomonas* spp. observed by analysis of fecal pellet DNA from *E. muris*-infected, naturally colonized hosts (Fig. 3). Notably, fecal copy number did not predict cecal burden. Similarly, *E. muris* levels were reduced in cecal content in co-infected mice, and fecal pellet copy number was not significantly different (Fig. 4B).

Given the consistent persistence of *Tritrichomonas* in this simultaneous co-infection model, we asked whether the tuft cell-ILC2 circuit was activated in the small intestine. Due to the challenges associated with analysis of intestinal tissue with high levels of type 2 immune cell activity by flow cytometry (15, 38), we performed bulk RNA-seq on minimally processed distal ileum from uninfected JAX mice, JAX mice infected with *E. muris* or *Tritrichomonas* spp. alone, and co-infected mice at 28 dpi. Principal component analyses (PCAs) and hierarchical clustering analysis on the top differentially expressed genes demonstrated that infection with *Tritrichomonas* spp. alone and animal sex were significant drivers of gene expression (Fig. 4C; Fig. S4A). *E. muris*-infected mice clustered together with uninfected controls (Fig. 4C; Fig. S4A). Essentially, no genes in ileal tissue from *E. muris-*infected mice were significantly different from uninfected control mice (Table S1, adjusted *P*-value cutoff < 0.10). Co-infected males and females clustered separately (males) or together (females) with mice from the same sex infected with *Tritrichomonas* spp. alone (Fig. S4A), suggesting sex impacts tissue response to co-infection.

Among the differentially expressed genes between the ileum from *Tritrichomonas-*infected mice and uninfected controls (Table S1), tuft cell genes (including *Pou2f3*, the lineage-defining transcription factor for tuft cells, and *Sucnr1*, the succinate receptor) and type 2 immune-related genes were significantly higher in mice infected with *Tritrichomonas* spp. alone relative to all other groups (Fig. 4D). Although these transcripts were significantly lower in the co-infected group, some co-infected mice had relatively high levels of tuft cell-related transcripts, while some *Tritrichomonas-*infected mice had relatively low expression. When we plotted the estimated cecal burden of *Tritrichomonas* spp. vs *Pou2f3* and *Sucnr1* transcript expression determined by RNA-seq, we identified a linear correlation between calculated cecal *Tritrichomonas* burden and tuft cell-ILC2 circuit transcript expression (Fig. S4B, *R*^2^ > 0.5). Fecal copy number did not show a significant correlation (Fig. S4B).

*Tritrichomonas*-mediated induction of the tuft cell-ILC2 circuit leads to significant remodeling of the small intestine, including intestinal lengthening (15). Only JAX mice mono-infected with *Tritrichomonas* spp. had significantly longer small intestines relative to uninfected controls; co-infected mice did not have significant gut lengthening relative to uninfected controls (Fig. 4E). Lengthening in *Tritrichomonas*-infected mice was primarily observed in males (denoted as “x”), suggesting that some physiological outcomes of tuft cell-ILC2 circuit activity are sex specific.

We also examined the frequency of ileal tuft cells using immunofluorescence to corroborate our sequencing findings. To account for regional heterogeneity, we quantified total DCLK1^+^ tuft cells along 8 cm of distal small intestine using unbiased, spot-based counting (Fig. S4C). Although there was a trend toward increased tuft cells in JAX mice infected with *Tritrichomonas* alone compared to *E. muris* mono-colonized and uninfected mice, tuft cell count was highly variable using this unbiased approach (Fig. 4F). Sexual dimorphism was evident in these data: *Tritrichomonas*-infected female mice (closed circles) had an increase in total DCLK1^+^ epithelial cells that we did not observe in male mice.

These data further support that activation of the tuft cell-ILC2 circuit by *Tritrichomonas* spp. is reduced in the presence of *E. muris*, even when co-infection is performed simultaneously, and *Tritrichomonas* remains detectable in both cecal and fecal content. In both co-infected mice and mice infected with *Tritrichomonas* alone, tuft cell transcript levels in whole intestinal tissue correlate linearly with cecal *Tritrichomonas* abundance, suggesting that the primary impact of *E. muris* in our co-infection model is in reducing total *Tritrichomonas*. However, the correlation between *Tritrichomonas* burden and tuft cell count (as determined by imaging) or intestinal lengthening was obfuscated by sex-divergent phenotypic responses to *Tritrichomonas* spp. infection, which is not evident at the transcriptional level.

### Co-infection with *E. muris* does not reduce succinate levels in cecal content

**Fig 5:**
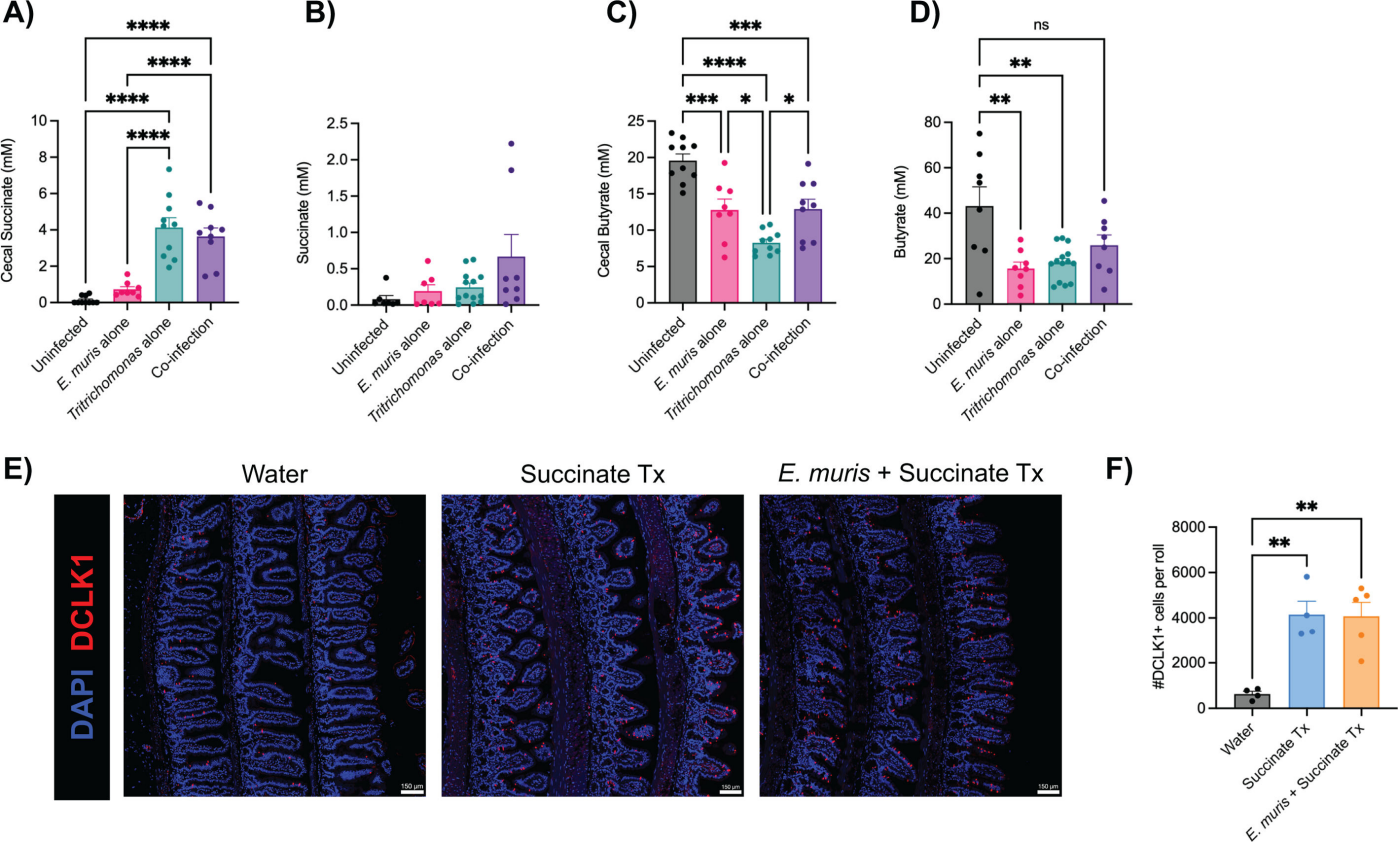
Total cecal succinate is not reduced during co-infection. (**A–D**) Quantitation of short-chain fatty acids (SCFAs) by GC/MS of extracted, whole cecal material (cecal) or clarified cecal supernatant. (**A and B**) Succinate. (**C and D**) Butyrate. (**E**) Representative images of distal small intestine Swiss rolls stained with an antibody against DCLK1 (red) to identify tuft cells from mice supplemented with succinate for 28 days in the presence or absence of *E. muris*. (**F**) Tuft cell (DCLK1 staining) quantification in the distal small intestine of mice treated with succinate with and without *E. muris*. (**A–D and F**) Error bars represent the standard error of the mean (SEM). Ordinary one-way analysis of variance followed by Tukey’s multiple comparisons. ns, not significant; *P < 0.05, **P < 0.005, ***P < 0.0005, and ****P < 0.0001.

Given the correlation between *Tritrichomonas* spp. levels in the cecum and activation of the tuft cell-ILC2 circuit in the small intestine, we speculated that cecal succinate levels would be reduced in co-infected mice relative to mice infected with *Tritrichomonas* spp. alone. To this end, we harvested whole cecal contents for mass spectrometry-based quantification of succinate and related metabolites. As previously demonstrated (15, 16), succinate in total cecal content was significantly increased in *Tritrichomonas*-infected mice compared to uninfected mice and mice infected with *E. muris* alone (Fig. 5A). Contrary to our hypothesis, co-infected mice had equally elevated levels of cecal succinate. Correlation between cecal succinate and calculated cecal *Tritrichomonas* burden was weak (Fig. S5A, *R*^2^= 0.22). Analysis of centrifugal-clarified cecal supernatant, free of protist trophozoites, showed no statistically significant differences among any experimental groups, suggesting that differential secretion into the soluble cecal fraction also does not explain the level of tuft cell-ILC2 circuit activity (Fig. 5B).

Recent work suggests that butyrate inhibits tuft cell differentiation (19). Butyrate levels in cecal content were significantly decreased in mice infected with any protist, with the lowest concentrations detected among *Tritrichomonas* spp. mono-colonized mice (Fig. 5C). Levels of butyrate in clarified cecal supernatant were significantly higher in control mice compared to mice infected with *E. muris* or *Tritrichomonas* spp. alone but not compared to co-infected mice (Fig. 5D), perhaps indicating that negative regulation of the tuft cell-ILC2 circuit by butyrate is increased in mice co-infected with *E. muris*. In contrast to previously observed increases in cecal acetate levels among germ-free mice infected with *Tritrichomonas* spp. (15), we did not observe any change in acetate concentrations in protist-colonized specific pathogen-free (SPF) mice (Fig. S5B). The related short-chain fatty acid (SCFA), propionate, was elevated across all protist-infected groups (Fig. S5B). Among the amino acids included in our metabolic panel, we observed a significant increase in glycine and proline concentrations in the cecal content of mice infected with *Tritrichomonas* spp. alone, which has not previously been reported (Fig. S5C). These data suggest that levels of butyrate, glycine, and proline are normalized in co-infected mice relative to mice colonized with *Tritrichomonas* alone, which could correlate with failure to activate the tuft cell circuit, active *E. muris*-mediated induction of negative regulation on tuft cell differentiation, or impacts of either protist on the bacterial microbiome.

Given the high levels of cecal succinate observed in co-infected mice, we considered whether *E. muris* might alter the sensitivity of intestinal tuft cells to succinate. To test this, we treated mice infected with *E. muris* cysts with succinate in the drinking water for 4 weeks. All mice receiving succinate-treated drinking water had tuft cell hyperplasia, whether they were infected with *E. muris* or not (Fig. 5E), suggesting that *E. muris* colonization does not actively prevent succinate-mediated intestinal remodeling. However, this level of succinate exceeds physiological levels observed in the cecum of *Tritrichomonas*-colonized mice and, when provided in the water, leads to rapid activation of the tuft cell-ILC2 circuit along the length of the small bowel.

As tuft cell activation occurs in the distal small intestine, not the cecum, where we measured succinate and *Tritrichomonas* burden, we considered whether ileal *Tritrichomonas* could be measured and correlated with tuft cell activation. However, quantification of *Tritrichomonas* from distal small intestine contents from naturally colonized mice by qPCR frequently failed (4 of 11 samples), and the successful amplifications from the ileum were highly variable (Fig. S5D and E). Ileal *Tritrichomonas* burden correlated poorly with cecal burden, which our data suggest is a robust predictor of tuft cell activation, suggesting limited utility of this approach. While this does not rule out ileal succinate being specifically lost during co-infection, metabolomics could not be attempted on ileal content due to technical limitations.

### *Tritrichomonas* spp. induce widespread colonic transcriptional changes, which are muted by *E. muris* co-infection

**Fig 6:**
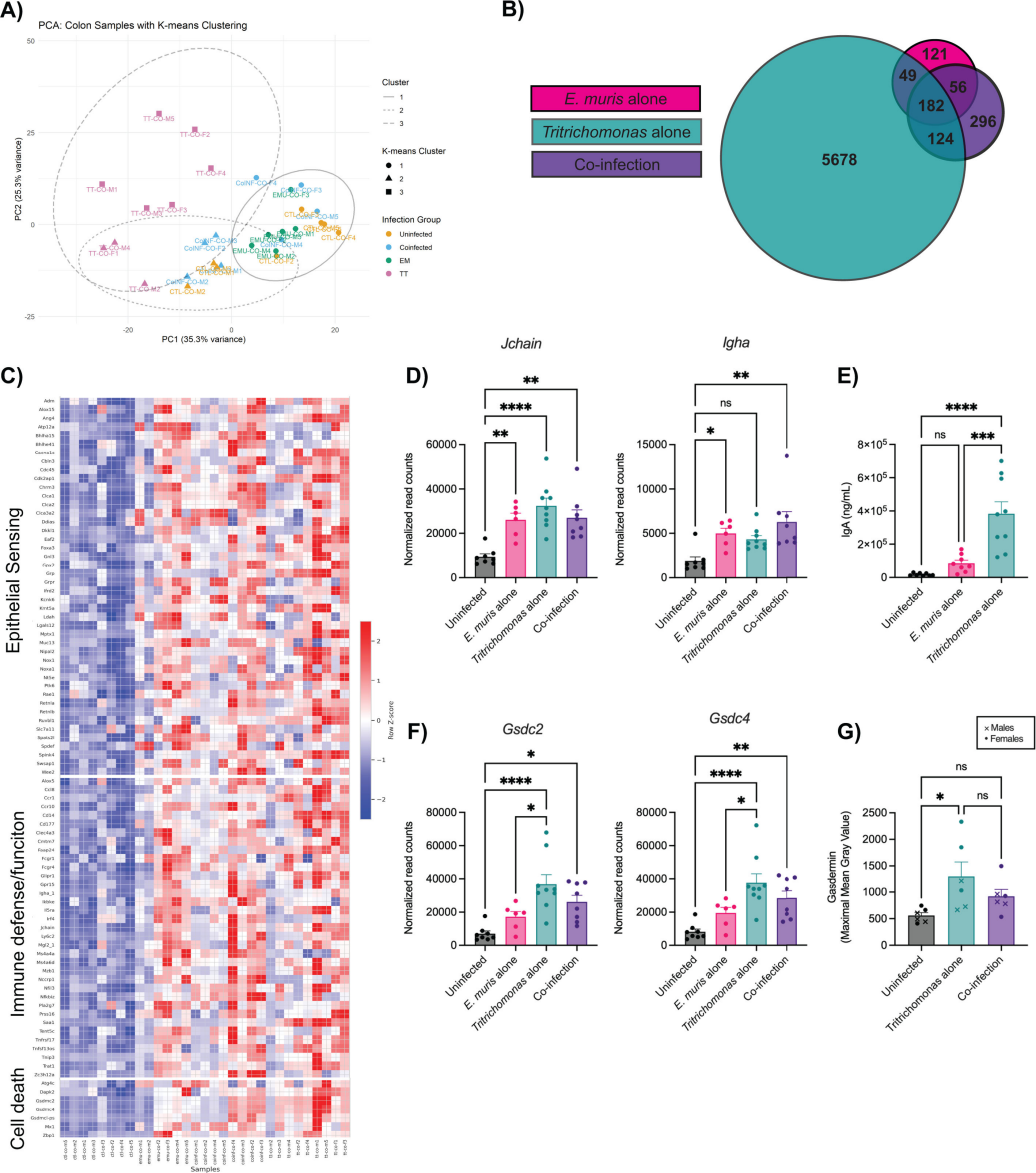
*Tritrichomonas* spp. induce widespread transcriptional changes in colon which are muted in *E. muris* co-infection. (**A–G**) JAX mice co-infected with *Tritrichomonas* and *E. muris* as described in Fig. 4A. (**A**) PCA of transcripts from bulk-RNA sequencing of the proximal colon. (**B**) Number of differentially expressed genes *increased* relative to uninfected controls from *E. muris* alone, *Tritrichomonas* alone, and co-infection groups. (**C**) Heatmaps of shared upregulated genes from protist-infected mice grouped by function. (**D**) *Jchain* and *Igha* expression by bulk RNA-seq. (**E**) Serum IgA levels quantified by ELISA. (**F**) *Gsdc2* and *Gsdc4* expression by bulk RNA sequencing. (**G**) Quantification of Gasdermin intensity in apical colon epithelium from mice stained with an antibody against Gsdmc2/Gsdmc3. (**D–F**) Error bars represent the standard error of the mean (SEM). Ordinary one-way analysis of variance followed by Tukey’s multiple comparisons. ns, not significant; *P < 0.05, **P < 0.005, ***P < 0.0005, and ****P < 0.0001.

Along with activation of the tuft cell-ILC2 circuit in the small intestine, infection with *Tritrichomonas* spp. induces robust changes in the colon mucosal immune environment (21–23). As *Tritrichomonas* and *E. muris* are abundant in the hind gut, we examined the consequences of single vs co-infection using RNA-seq analysis of minimally processed proximal colon tissue. As in our ileal RNA-sequencing analysis, PCA and hierarchical clustering of colonic transcriptomes demonstrated sex and infection differences, with *Tritrichomonas* spp. infection driving the most significant transcriptomic shifts (Fig. 6A and B; Fig. S6A).

In contrast to the ileum, we observed differentially expressed transcripts in all experimental groups relative to uninfected mice (Table S2, adjusted *P* value < 0.10). Examining the overlap of differentially expressed genes increased in all protist-infected mice relative to uninfected controls, we identified a small set of transcripts upregulated in the colon in all infection conditions relative to uninfected mice (182 genes, 167 with normalized average read count >20; Fig. 6B and C; Fig. S6B; Table S3). Many of these transcripts related to immune function, epithelial cell activation, and cell death (Fig. 6C). Two specific gene signatures that were upregulated in all groups have previously been described in the context of *Tritrichomonas* colonization and/or elevated systemic type 2 immune responses—increased IgA^+^ plasma cells and increased expression of gasdermins. These results are an example of tissue-specific host responses in which *E. muris–Tritrichomonas* competition does not markedly alter protist abundance, yet they reshape the host transcriptional landscape.

*Tritrichomonas* colonization is associated with non-specific increases in mucosal and systemic IgA (25). Our sequencing data suggested that this could also be true for *E. muris* (Fig. 6D). However, when we examined serum IgA following *E. muris* infection, it was not significantly increased over controls. As previously described (25), natural *Tritrichomonas* colonization was associated with elevated serum IgA (Fig. 6E). While this does not exclude local induction of IgA responses by *E. muris*, it suggests that *E. muris* does not induce a rapid rise in systemic IgA.

Increased expression of gasdermins has been observed in small intestinal epithelial cells during helminth infection and chronic type 2 cytokine exposure (29, 40–43). We also observed this increased expression in the ileum of *Tritrichomonas*-infected mice (Fig. S6C). However, this has not been examined in colonic epithelium. In the colon, we found that the *Gsdmc2* and *Gsdmc*4 were upregulated transcriptionally in all protist-infected mice (Fig. 6F). To address this further, we stained for Gsdc2/c3 proteins in the colon. We observed robust protein signals in colon epithelial cells from *Tritrichomonas-*infected mice (Fig. 6G; Fig. S6D and E). This, however, was absent in our other experimental groups. Once again, we observed sexual dimorphism, which was not evident transcriptionally, with females more robustly upregulating Gsdc2/c3 expression in response to *Tritrichomonas* spp. mono-infection compared to males (Fig. 6G; Fig. S6E).

## DISCUSSION

Here, we have explored the immunological consequences of infection with *Entamoeba muris*, as well as how *E. muris* impacts the immune response induced by *Tritrichomonas* spp. *E. muris* infection did not elicit significant immune responses in the gut in C57BL/6J mice, with strikingly little impact noted specifically in the small intestine. Recent studies conducted in BALB/c mice found that *E. muris* infection resulted in colitis-like pathology (11). Since we did not observe these changes in colon from *E. muris*-infected in-house or vendor-derived C57BL/6J mice, it is possible that genetic differences between these strains influence the immune response to *E. muris*, as previously seen for succinate-driven tuft cell responses (44). The lack of immune cell recruitment or epithelial remodeling observed in our studies is particularly striking in the context of two different models of co-infection with *Tritrichomonas* spp. Introduction of *E. muris* into mice stably colonized with *Tritrichomonas* spp. led to a rapid reduction of *Tritrichomonas* spp. burden and loss of tuft cell activation, while in our simultaneous co-infection model, persistent *Tritrichomonas* nevertheless failed to robustly activate tuft cells. This appears to be largely through reduction of *Tritrichomonas* burden, as we observed a linear relationship between cecal *Tritrichomonas* burden and measures of tuft cell-ILC2 circuit activation. However, we did not detect a reduction in cecal succinate in co-infected mice, despite reduced cecal *Tritrichomonas*.

Our data suggest several models whereby co-infection with *E. muris* negatively impacts the ability of succinate-producing *Tritrichomonas* to persistently activate tuft cells. First, tuft cell activation occurs in the distal small intestine, not the cecum, where we measured both succinate (which failed to correlate with tuft cell-ILC2 circuit activity in the distal small intestine) and *Tritrichomonas* load. It is likely that local, ileal succinate is critical for tuft cell activation, and this may be specifically lost in the presence of *E. muris* in a way that was not tested in our experiments. Second, *E. muris* could directly or indirectly inhibit type 2 cytokine-driven tuft cell specification and expansion, perhaps by inducing HDAC3 antagonists (i.e., butyrate [19, 45]) or reducing signaling pathways critical for driving tuft cell hyperplasia (i.e., IL4Rα, KIT [17, 46]). As others have observed long-term persistence of the immune and epithelial remodeling induced by tuft cell activation even months after the activating stimulus is withdrawn (43), the loss of tuft cell-ILC2 circuit activity after *E. muris* introduction suggests that reduction in *Tritrichomonas* burden alone would be insufficient to extinguish this type 2 immune circuit after 4 weeks, indicating that both loss of signal and active repression may be at play in co-infection. Overall, our data suggest that indirect competition between *E. muris* and *Tritrichomonas* spp. ultimately reduces total *Tritrichomonas* burden and/or succinate secretion, perhaps leading to reduced local succinate in the distal small intestine and extinguishment of tuft cell activation.

Related to both models, the known roles of *Entamoeba* (a bacterial predator) and *Tritrichomonas* in altering the bacterial microbiome (8, 12, 16, 26, 29) raise the possibility that succinate-mediated tuft cell activation and *Tritrichomonas* burden depend on a permissive bacterial microbiome, which is likely profoundly altered by the introduction of another protist. The divergent impacts on *Tritrichomonas* spp. burden in our two different models of co-infection strongly suggest bacterial microbiome impacts *Tritrichomonas* burden. We speculate that the introduction of *E. muris* into mice naturally colonized with *Tritrichomonas* spp*.*, in which *Tritrichomonas* is likely a keystone species, may have led to the ecological collapse of bacterial communities that rely on, and possibly also sustain, *Tritrichomonas*. In contrast, simultaneous co-infection of mice that are protist-naïve may create a more favorable environment for the establishment of both phyla, though the level and the ability of *Tritrichomonas* to activate tuft cells in this context may be limited. Additional evidence suggests the role of the bacterial microbiome in shaping protist immune response. While we observed a trend in the distal small intestine toward a type 17 immune response upon *E. muris* infection (Fig. 1), this was not seen when we performed *E. muris* single infection into JAX mice. Changes to the bacterial microbiome with natural colonization and introduction of the protists will be an avenue of future studies.

Although we observed a correlation between cecal *Tritrichomonas* spp. level and tuft cell transcripts in the distal small intestine, we did not observe this correlation with cecal succinate levels. Notably, our cecal succinate testing captures both secreted succinate from species like *T. musculus*, which activates tuft cells, and intracellular succinate from mucin-consuming species like *T. casperi*, which does not activate the tuft cell-ILC2 circuit (16). While our data suggest that more than one tritrichomonad species exists within our colony, we were unable to quantify an enrichment specifically of *T. casperi* in co-infected mice due to technical limitations. Alternatively, our imaging suggests that persistent *Tritrichomonas* in co-infected mice may be in a pseudocyst form (26), which would not actively secrete succinate. Intracellular succinate in *Tritrichomonas* pseudocysts has not been quantified.

In addition to identifying that co-infection with *E. muris* leads to rapid loss of the type 2 immune response activated by *Tritrichomonas*, our quantification of tuft cell-ILC2 activation in the presence of *Tritrichomonas* suggests several previously unappreciated aspects of this circuit. Our data indicate that fecal pellet surveillance for *Tritrichomonas* does not reflect cecal burden and is likely a poor predictor of tuft cell-ILC2 circuit activation in the distal small intestine, particularly if screening is not performed for other protists. Fecal *Tritrichomonas* abundance may be more representative of stress or niche capacity and may be particularly poor at detecting mucosal-associated protists (39). However, we show that transcriptional evidence of tuft cell-ILC2 circuit activation in the distal small intestine can be predicted based on the level of *Tritrichomonas* burden in the cecum. We also identified biological sex as a critical determinant in either the magnitude of type 2 immune cell activation in *Tritrichomonas-*colonized mice or the downstream physiological impacts of type 2 immune activity on intestinal physiology, such as the magnitude of tuft cell expansion and gut lengthening. This finding aligns with other reports of sexual dimorphism in ILC2 function and IL-13-mediated signaling (47–49).

Our findings provide novel insight into the host response to *E. muris* colonization, including identification that this relatively immunologically “silent” protist can profoundly alter the ability of *Tritrichomonas* spp. to exert immunomodulatory effects. These findings broaden our understanding of inter-protist interactions in the gut and underscore the importance of considering cross-eukaryotic dynamics when studying host-microbiome-immune interactions.

## MATERIALS AND METHODS

### Animal husbandry

All mice used throughout this study were 8–12 weeks old at the time of oral challenge. Males and females were equally matched in all groups.

### Protist isolation and purification

*E. muris* cysts were isolated using sucrose gradients from fecal samples of naturally colonized C57BL/6 mice, as previously described (10). Fecal samples were ground, homogenized, filtered through gauze, centrifuged, resuspended in water, and layered over 1.5 M sucrose. The cyst-containing aqueous mid-layer was washed and concentrated into a cyst-containing pellet. Cyst isolation was also used for quantifying infectious material from fecal pellets in Fig. 1.

Isolation of *Tritrichomonas* spp. trophozoites for oral infection was performed as previously described (15). Mouse ceca were harvested from in-house bred, naturally colonized C57BL/6 mice. Cecal suspensions were filtered, centrifuged, and washed in sterile PBS twice. *Tritrichomonas* spp. trophozoites were counted on a hemocytometer. For experiments utilizing mice sourced from Jackson Laboratories, 1× Pen-Strep (Sigma-Aldrich, cat. P4333) was added to each PBS wash.

### Mouse infections and treatments

#### *E. muris* colonization of in-house mice

C57BL/6 Flare25-reporter mice (17), maintained in-house with or without *Tritrichomonas* spp., were used as recipients of *E. muris*. Mice were gavage-fed with either purified *E. muris* cysts (between 2.5 × 10^4^ and 1.5 × 10^5^) or 1× PBS as a control. We confirmed *Entamoeba* and *Tritrichomonas* status in these animal colonies by 18S PCR and visual cecal content inspection prior to infection.

#### Primary *E. muris* and *Tritrichomonas* infections of protist-free mice

Male and female C57BL/6 mice were purchased from Jackson Laboratories (room EM09) and regularly screened for the following protists: *Cryptosporidium* spp., *Giardia muris*, *Entamoeba muris*, *Spironucleus muris*, and Trichomonads: *Tritrichomonas muris* and *Tritrichomonas minuta. Tritrichomonas* recipients (mono- and co-infected) were gavage-fed 1.5 × 10^4^ *Tritrichomonas* spp. trophozoites. *E. muris* recipients (mono- and co-infected) were gavage-fed 5 × 10^3^ *E. muris* cysts. Control mice received the same volume of 1× PBS by oral gavage.

#### Succinate treatment

Male and female wild-type C57BL/6 mice were purchased from Jackson Laboratories and gavaged with purified cysts (7 × 10^4^) or left unmanipulated as uninfected controls. Succinate-treated mice received 100 mM succinic acid, pH 7.1 (Sigma-Aldrich) via drinking water *ad libitum* for 28 days, while untreated mice were administered untreated drinking water adjusted to pH 7.1.

### Genomic DNA extractions

For experiments in Fig. 1 to 3, gDNA from mouse fecal pellets was isolated as described previously (50, 51) with the following modifications. Whole feces (~0.10 g) were placed in solvent-resistant screw-cap tubes containing 0.1 mm zirconia/silica beads (BioSpec Products 11079101z) and one large stainless steel bead (BioSpec Products 11079132ss), suspended in 500 µL lysis buffer (200 mM Tris-HCl, pH 8.0, 200 mM NaCl, and 20 mM EDTA), 210 µL 20% SDS, and 500 µL UltraPure phenol/chloroform/isoamyl alcohol, pH 7.9, 25:24:1 (Invitrogen 15593-049). Fecal samples were beaten for 3 min at room temperature using a 115-volt MiniBeadbeater-96 (BioSpec Products), and gDNA was precipitated with 3 M sodium acetate and 100% isopropanol overnight. gDNA was washed with 100% ethanol and concentrated into TE buffer using the DNA Clean and Concentrator 5 kit (Zymo Research D4004).

In co-infection experiments in Jax mice, gDNA was isolated from ~150 mg of fecal pellets or cecal content using QIAamp Fast DNA Stool Mini Kit (Ref 51604) with additional pellet homogenization before extraction: pellets were transferred to tubes containing 1.5 mm zirconium beads (Benchmark Scientific, D1032-15) with 1.5 mL InhibitEX Buffer and homogenized using a microtube homogenizer (Bead Bug) at maximum speed for 30 s. Suspensions were heated at 95°C for 5 min and then homogenized a second time using the same conditions. All gDNA was quantified using a Nanodrop One spectrophotometer.

For small intestine (3 cm distal ileum sans 1 cm of cecal junction) protist quantitation, the intestinal content was flushed with sterile PBS into a sterile tube, then spun at max speed for 5 min to pellet the content, which was then resuspended in 1 mL InhibitEX buffer before starting homogenization.

### Protist DNA analysis by PCR and qPCR

For 18S qPCR detection of *E. muris* and *Tritrichomonas* spp. in fecal pellets and cecal contents, standard curves were generated using two methods, noted in figure legends and text. In method 1, isolated protists or cysts counted via hemocytometer were lysed using QIAamp Fast DNA Stool Mini Kit (see above). gDNA was serially diluted to create a standard curve of protist/cyst equivalents. In method 2, synthetic standards were generated via 2 rounds of PCR targeting *Entamoeba* 18S or *Tritrichomonas* 18S (BIONEER, AccuPower Taq PCR Premix, 8 reactions pooled) and agarose gel purification using the QIAquick Gel Extraction kit (Qiagen, cat. 28704). The purified DNA was quantified on a Nanodrop One (Thermo Fisher) spectrophotometer and converted to copies/µL (660 daltons per base pair). Amplicons were serially diluted (1 × 10^7^ to 1 genome equivalents) to generate *Entamoeba* 18S and *Tritrichomonas* 18S standard curves.

For 18S qPCR detection and amplicon purification, previously published primers were used.

Pan-*Entamoeba* primers (10) were as follows: Forward, 5′-TCG AGA TAA ACG AGA GCG AAA G-3′, and Reverse, 5′-GTC AGG ACT ACG ACG GTA TCT A-3′. For 18S qPCR detection of *Tritrichomonas*, the previously published *Tritrichomonas* qPCR primers were used (15): Forward, 5′-AGA GGA AGG AGA AGT CGT AAC AAG G-3′; Reverse, 5′-CTC GTG TAA GAA GCC AAG ACA TCC-3′.

For end point PCR and qPCR of *Tritrichomonas* species, gDNA was isolated from whole cecal content and amplified using published PCR primers (16): *T. casperi* Forward, 5′-AGG TTA CTG AAT CAT ACA TGC GT-3′, and Reverse, 5′-GCA GGA GTT GCT TTC ATT GTG-3′; *T. musculus* Forward, 5′-GCT TTT GCA AGC TAG GTC CC-3′, and Reverse, 5′-TTT CTG ATG GGG CGT ACC AC-3′.

### Quantification of cecal content and supernatant SCFAs by GC-MS

Whole cecal contents were either snap frozen or extruded into microcentrifuge tubes and centrifuged to isolate clarified cecal supernatant. Sample processing and analysis were performed by the Host-Microbe Metabolomics Facility at the University of Chicago. Complete protocols and equipment lists are available at https://dfi.uchicago.edu/host-microbe-metabolomics-facility/hmmf-methods-and-resources.

### Intestinal tissue harvesting, fixation, and sectioning

Small intestines were dissected from the pyloric sphincter to the ileocecal junction and suspended alongside a vertically positioned measuring tape to avoid stretching or tearing of the tissue. Total intestinal length was recorded in centimeters. Whole colons and ceca were dissected and fixed in anhydrous methacarn fixative (60% methanol, 30% chloroform, and 10% glacial acetic acid) for 16–24 h at 4°C. For intestinal Swiss rolls, 10 cm of the distal mouse small intestine was cut from the cecum. Two centimeters proximal to the cecum was removed for a total of 8 cm of distal small intestine that was flushed with modified Bouin’s fixative (50% ethanol and 5% glacial acetic acid) and 1× PBS. Following this, intestines were Swiss rolled and fixed in 10% neutral buffered formalin for 16–24 h at 4°C (52). Tissues were rinsed and stored in 70% ethanol before paraffin embedding and sectioning by the Wisconsin-Madison Carbone Cancer Center Experimental Animal Pathology Laboratory, which also performed H&E staining using standard protocols.

### Tissue immunofluorescence staining and imaging

Tissue slides were baked at 60°C for 1 h. Slides were deparaffinized by three 10 min xylene washes (Sigma-Aldrich), followed by two changes of absolute ethanol, two changes of 95% ethanol, and one change of 70% ethanol, each for 1 min. After rinsing with Nanopure water, slides were transferred into antigen retrieval buffer (10 mM Tris, 1 mM EDTA, 0.05% Tween 20, pH 9.0) and placed into a pressure cooker at low pressure for 1 min. Slides were cooled for 5 min in 1× PBS. Tissues were blocked for 1 h at room temperature using Perm/Block (0.3% Triton X-100 and 1% BSA in 1× PBS) solution, then incubated with primary antibodies diluted in Perm/Block overnight at 4°C in the dark. Tissues were washed three times in 1× PBS, then incubated with secondary antibodies diluted in Perm/Block solution for 2 h, followed by washing. Tissues were then blocked with 10% normal sera of the secondary antibody host species for 1 h at room temperature, then incubated with conjugated antibodies diluted in Perm/Block for 2 h. After washing, tissues were counterstained with 4′,6-diamidino-2-phenylindole dihydrochloride (DAPI) diluted in Perm/Block and washed before a coverslip was attached with ProLong Glass Antifade Mountant (Thermo Fisher Scientific). The mounting media were allowed to cure overnight. Tissue images were acquired on a Nikon A1R (20× or 40× objective, 0.80 NA) and analyzed with Imaris version 10.X (Bitplane).

#### Antibodies

Antibodies were as follows: anti-DCAMKL-1 (Abcam, ab31704, 1:50), anti-GSDMC2/GSDMC3 antibody (EPR20890-48; Abcam, ab229896, 1:500), polyclonal rabbit anti-*Tritrichomonas* (16, 1:100), donkey anti-rabbit IgG (H + L), Alexa Fluor 546 (Thermo Fisher, A10040, 1:250), wheat germ agglutinin-Oregon Green 488 (Invitrogen, W6748, 1:100), and anti-CD45 (BioLegend, 103108, 1:50).

#### Tuft cell quantification by imaging

Tuft cells (DCLK1^+^) were identified and quantified using the “Spots” function within Imaris. This function uses a predefined diameter for the fluorescent signal and marks it with a dot for quantification and visualization. A diameter of 20 μm was established to identify DCLK1^+^ cells based on the average diameter of randomly selected DCLK1^+^ signals across images. Non-specific background artifacts identified by the Spots function, particularly ones outside of the crypt-villus unit, were excluded manually.

#### Gasdermin intensity quantification

All colon tissue sections analyzed contained fecal pellets to preserve structure. The intensity of gasdermin staining was quantified in FIJI/ImageJ, utilizing the plot profile function on a line segment (width = 30). Each sample had six total line regions of interest (ROI) drawn across the apical epithelium—three on opposing sides of the lumen. The maximum intensity from each line ROI (six total) was averaged to generate the sample’s average maximal mean gray value.

### Intestinal dissociation and flow cytometry

Single-cell suspension of intestinal tissue was generated using previously published protocols (38). The “rapid” preparation was used for processing of all small intestinal tissues due to the known increased fragility and lower viability of *Tritrichomonas* spp.-infected tissues upon dissection. The “slow” preparation was used for processing all cecal and colonic tissue, as well as for small intestines analyzed in Fig. 1. Fc Block (Bio X Cell, 2.4G2) and antibodies to surface markers were diluted in FACS wash buffer (FWB, 2% fetal bovine serum, 0.1% sodium azide in 1× PBS) and added to single cell suspensions. Cells were washed and resuspended in FWB containing DAPI (Roche) for immediate analysis following staining, or fixed with 4% PFA for 2 min after staining with Live/Dead Violet fixable stain (Thermo Fisher Scientific, L34964 A) for later analysis. Cells for intracellular staining were stained with Live/Dead Violet fixable stain, and the FoxP3/Transcription Factor Staining Buffer Set (eBiosciences) was used according to the manufacturer’s instructions to analyze intracellular markers. Flow cytometric counting beads (CountBright Absolute; Life Technologies) were added for live cell gating and counts during acquisition.

#### Flow cytometry

Samples were analyzed on an LSR Fortessa (BD Biosciences) with five lasers (355 nm, 405 nm, 488 nm, 561 nm, and 640 nm). During analysis, FSC-H/FSC-A were utilized to select single cells, SSC-A and Live/Dead (DAPI or Live/Dead) were used to exclude dead cells, and FSC-A/SSC-A were used to exclude debris. Data were analyzed with FlowJo 10.8.2. The following flow cytometry antibodies were purchased from BioLegend: EpCam PerCP-Cy5.5 and BV711 (clone G8.8), Ly6G APCCy7 (1A8), CD11b BV711 (M1/70), CD45 BV785 (30-F11), Thy1 BV786 (30-H12), TCRγδ PerCPCy5.5 (GL3), NK1.1 PE-Cy7 (PK136), and NKp46/CD335 PE-Cy7 (29A1.4). Lineage-positive cells were excluded (L/D “Dump Gate”) in ILC staining using the antibodies in Pacific Blue, from BioLegend: CD11c (N418), F4/80 (BM8), CD11b (M1/70), FCeR1 (MAR-1), CD19 (6D5), B220 (RA3-6B2), Ter-119, and GR-1. T1/ST2 FITC/PE (DJ8) was purchased from MD Biosciences. The following antibodies were from eBiosciences/Invitrogen: GATA3 ef660 (TWAJ), TCRβ APC-ef780 (H57-597). The following antibodies were purchased from BD Biosciences: CD64a/b AF647 (X545/7.1), RORγT PE (B2D), CD45 BUV395 (30-F11), Siglec-F BB515 (E50–2440), and Thy1 BUV395 (3-H12).

### RNA extraction and library preparation

Approximately 3 cm long distal ileal and 2 cm proximal colonic tissue fragments were harvested beginning 1 cm upstream and downstream from each cecal junction. Tissues were immediately placed in Buffer RLT (Qiagen) on ice and homogenized using a gentleMACS Octo Dissociator (Miltenyi Biotec). RNA isolation and purification were performed using the RNeasy Mini Kit (Qiagen, cat. 74104), following the manufacturer’s instructions. Purified RNA was submitted to the University of Wisconsin-Madison Biotechnology Center’s Gene Expression Center for quality control analyses, RNA library preparation, sequencing, and read demultiplexing (RRID: SCR_017757). RNA integrity was assessed using an RNA ScreenTape assay on an Agilent 4200 TapeStation system. Bulk RNA library preparation was performed using the Illumina TruSeq Stranded mRNA kit for poly(A) enrichment. All samples were run in duplicate in two lanes of a 2 × 150 bp flow cell using the Illumina NovaSeq × Plus platform. Read quality was determined using FastQC (v0.11.9).

### RNA-seq data analyses

Raw FASTQ files were trimmed to remove adapter content, filter out low-quality reads, and group paired reads using Trimmomatic (v0.39). Trimmed reads were aligned to the *Mus musculus* strain C57BL/6 genome (GRCm38.p6, release 38; NCBI) using STAR (v2.7.5c) (53). Default parameters were used with the following exceptions: maximum mismatch (2 bp), minimum intron length (20 bp), and maximum intron length (100,000 bp). Quantification of mapped reads and counts table generation was performed using RSEM (v1.3.1) (54). Differential gene expression analyses were conducted using DESeq2 (55) in R.

Normalized read counts were extracted and used to compute the top 500 genes by variance, which were used to generate PCA plots and heatmaps. In heatmaps, RNA-seq read count matrices were variance-stabilized by computing row-wise *Z*-scores across all samples. Genes were grouped into functional categories based on annotation. Within each functional group in Fig. 6, genes were hierarchically clustered using average linkage on correlation distance. Columns (samples) were hierarchically clustered using Euclidean distance with Ward’s linkage. Heatmaps were plotted in R and Python (matplotlib/seaborn).

## Data Availability

All raw read sequences, processed data files, and metadata have been made publicly available via deposition into the Gene Expression Omnibus (GEO): accession number GSE320380.

## SUPPLEMENTAL MATERIAL

Figure S1 mbio.00802-26-s0001.pdf *E. muris* fecal cyst shedding and representative flow gating.

Figure S2 mbio.00802-26-s0002.pdf Loss of intestinal tuft cell-IL-25-ILC2 circuit activity in *Tritrichomonas* spp.-colonized mice infected with *E. muris*.

Figure S3 mbio.00802-26-s0003.pdf Species primer analysis and cecal imaging for persistent *Tritrichomonas* spp. after *E. muris* co-infection.

Figure S4 mbio.00802-26-s0004.pdf Sex-disaggregated transcriptional changes and *Tritrichomonas*-driven tuft cell hyperplasia in the small intestine of JAX mice infected with *Tritrichomonas* and *E. muris*.

Figure S5 mbio.00802-26-s0005.pdf Cecal metabolite shifts induced by protist infection and variability in terminal ileum detection of *Tritrichomonas* spp.

Figure S6 mbio.00802-26-s0006.pdf Colonic bulk RNA-seq suggests pan-protist changes in gene expression, but only *Tritrichomonas* infection upregulates gasdermin proteins.

Supplemental Legends mbio.00802-26-s0007.docx Supplemental figure and table legends.

Table S1 mbio.00802-26-s0008.xlsx Differentially expressed genes from RNA-seq of ileum.

Table S2 mbio.00802-26-s0009.xlsx Differentially expressed genes from RNA-seq of colon.

Table S3 mbio.00802-26-s0010.xlsx Upregulated genes in colon.

ASM does not own the copyrights to Supplemental Material that may be linked to, or accessed through, an article. The authors have granted ASM a non-exclusive, world-wide license to publish the Supplemental Material files. Please contact the corresponding author directly for reuse.
