# Supplementary figures and images for: Competition between commensal protists shapes gut mucosal immunity in mice
Source: mBio. 2026 May 18;17(6):e00802-26. doi: 10.1128/mbio.00802-26 (PMC13251391; doi:10.1128/mbio.00802-26)

A)

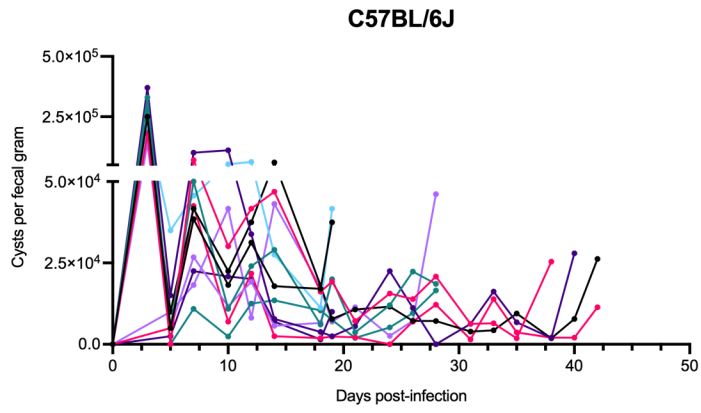

B)

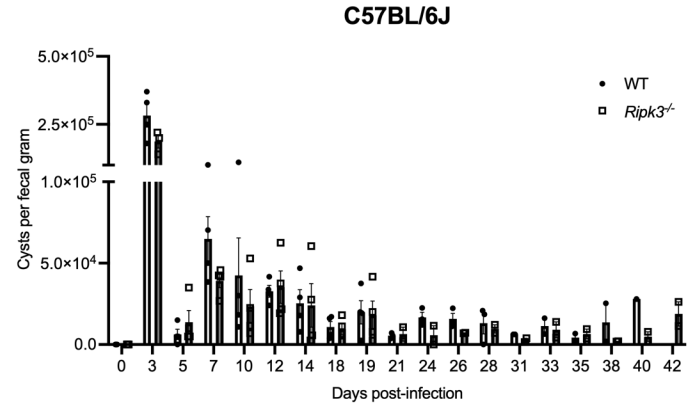

C)

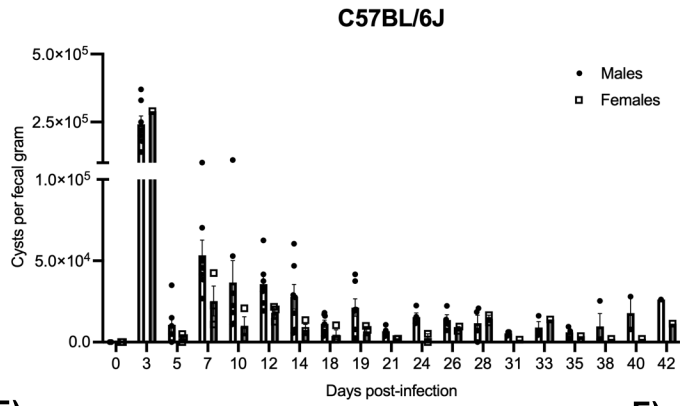

D)

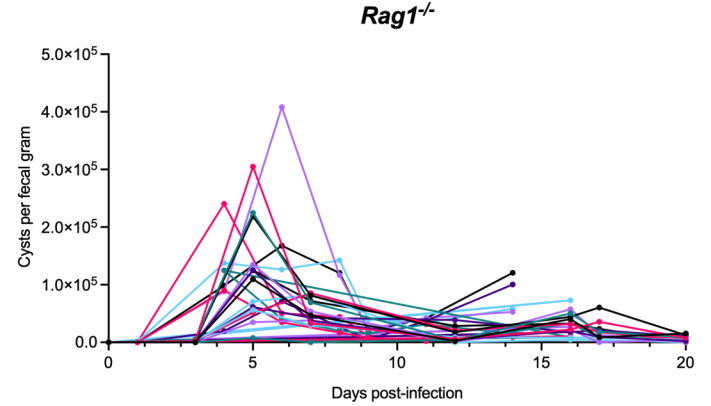

E)

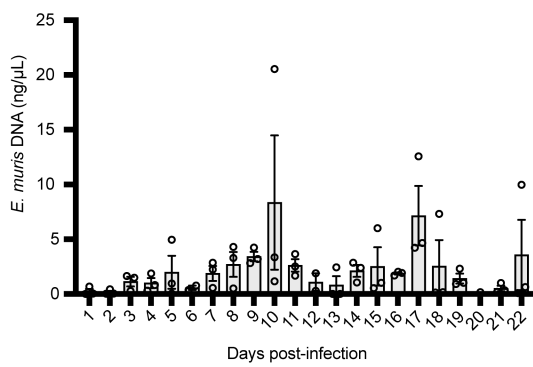

F)

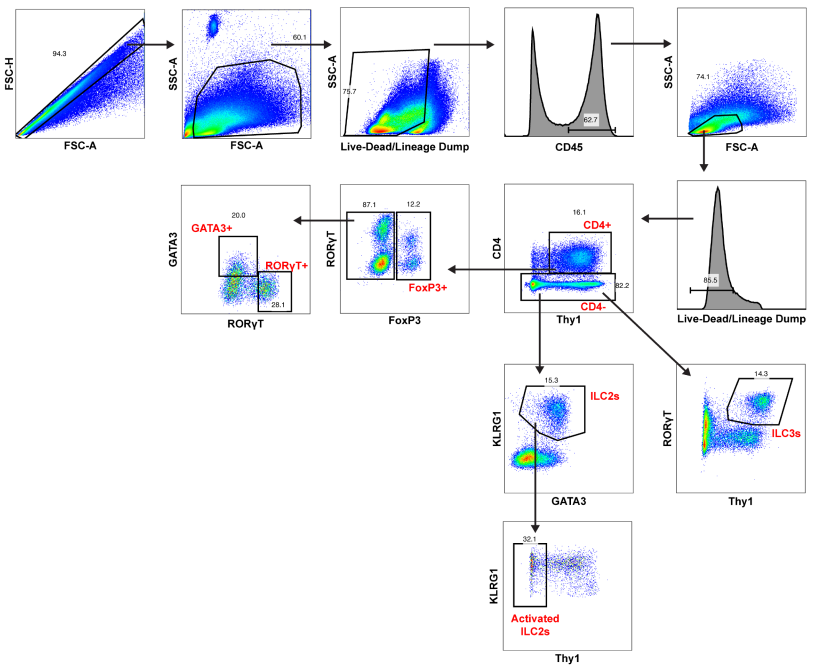

G)

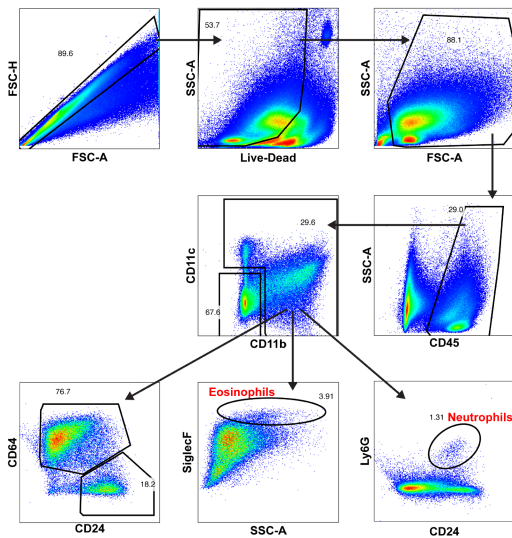

H)

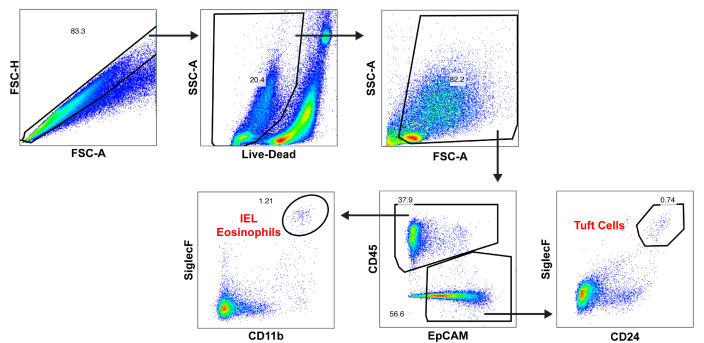

Supplement: Figure S1 — E. muris fecal cyst shedding and representative flow gating. [file mbio.00802-26-s0001.pdf]

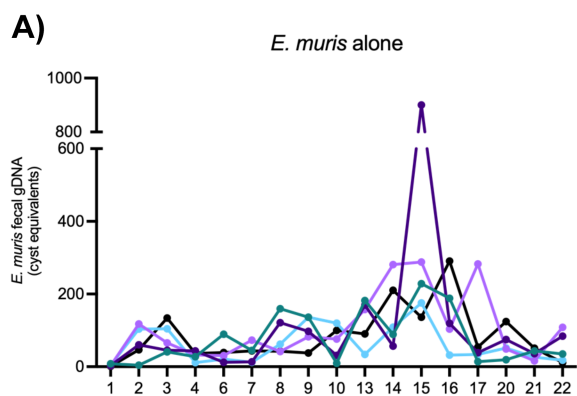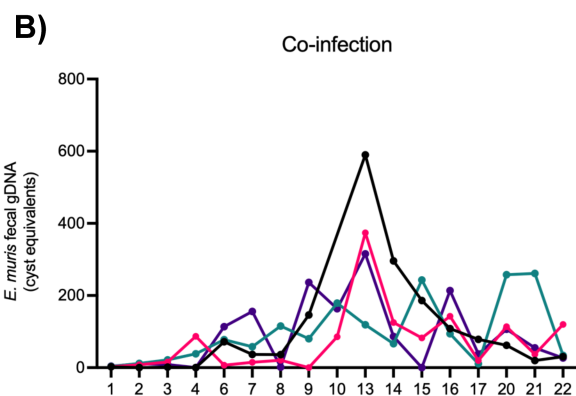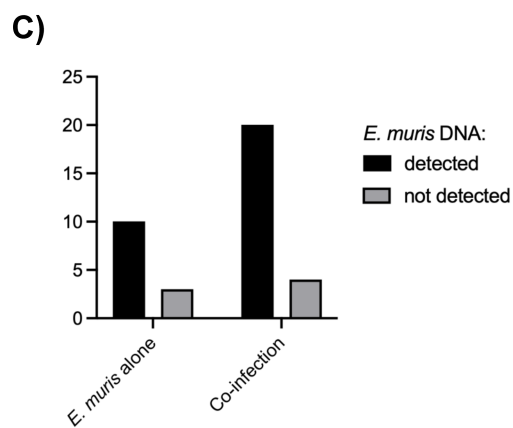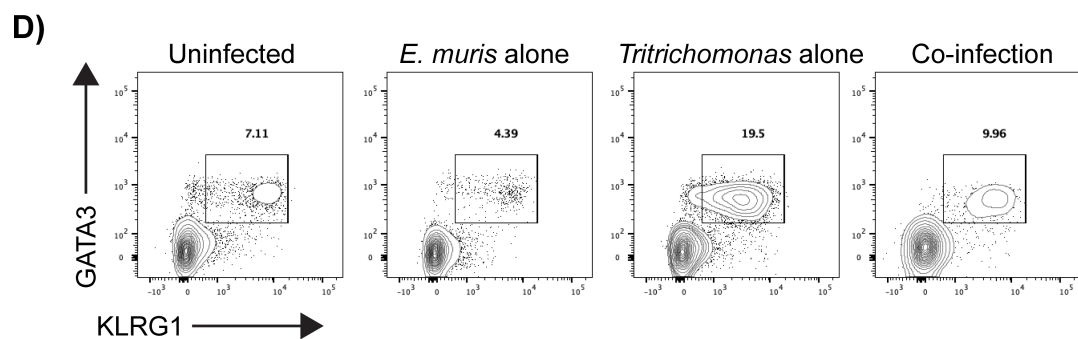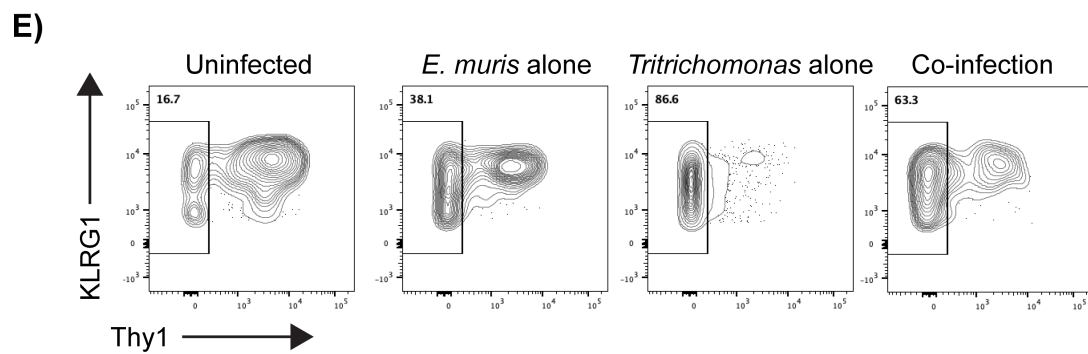

Supplement: Figure S2 — Loss of intestinal tuft cell-IL-25-ILC2 circuit activity in Tritrichomonas spp.-colonized mice infected with E. muris. [file mbio.00802-26-s0002.pdf]

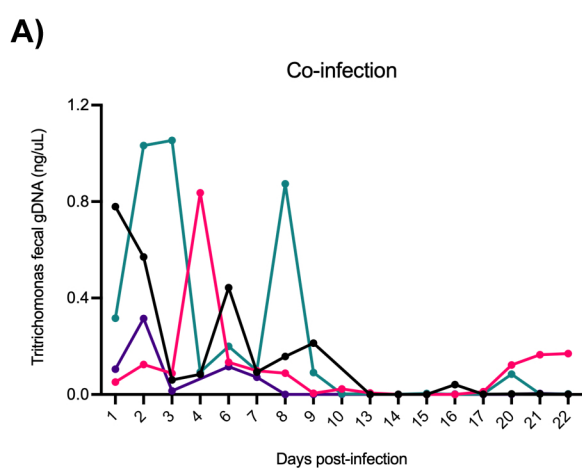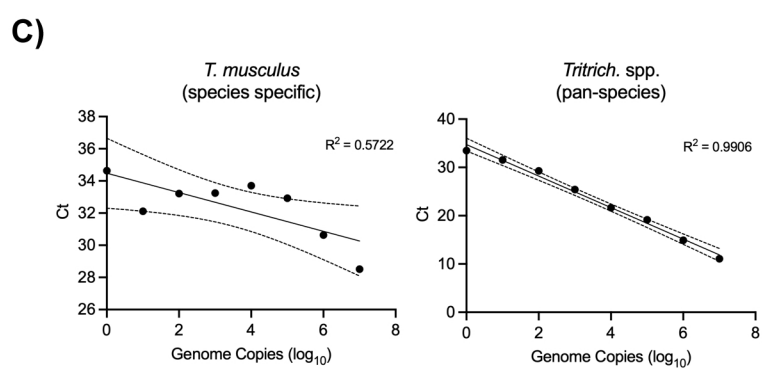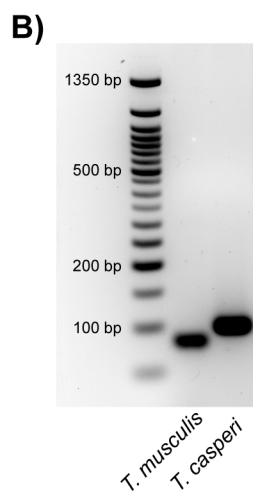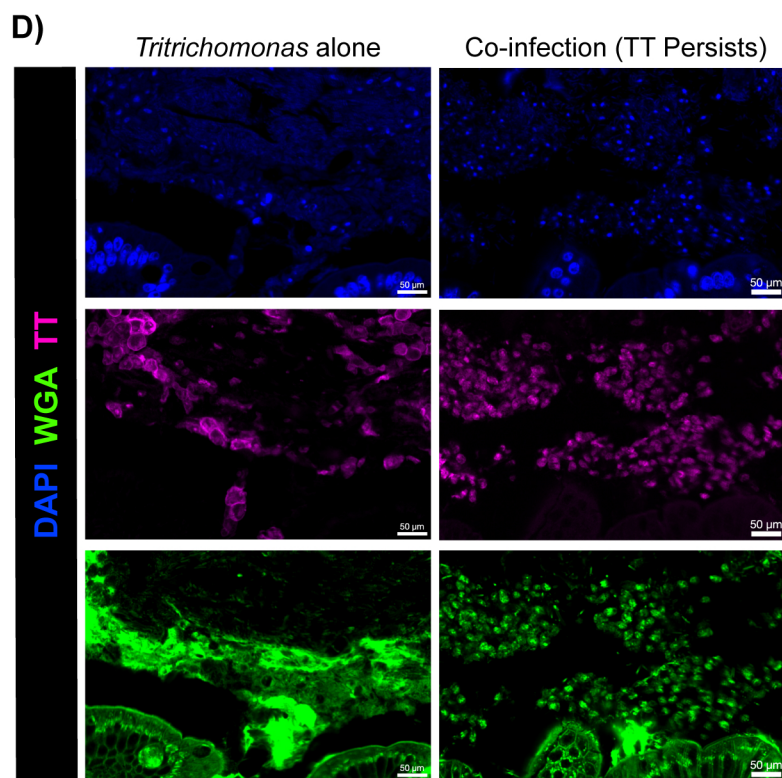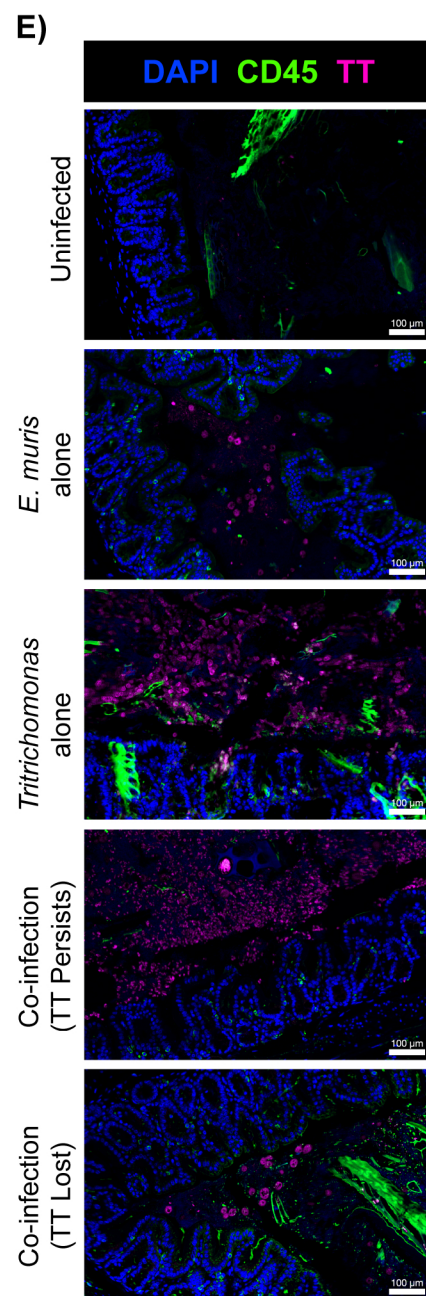

Supplement: Figure S3 — Species primer analysis and cecal imaging for persistent Tritrichomonas spp. after E. muris co-infection. [file mbio.00802-26-s0003.pdf]

A)

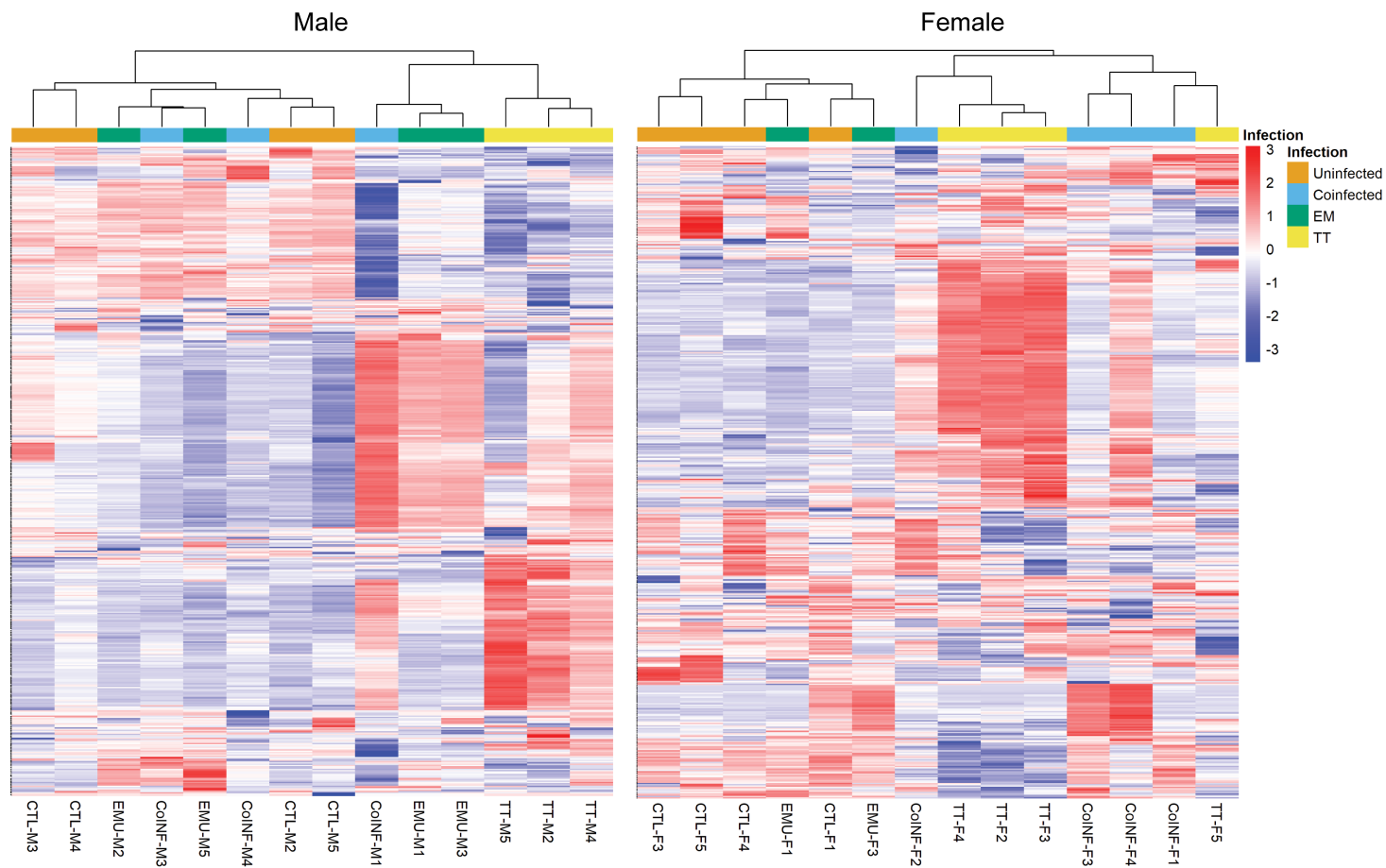

B)

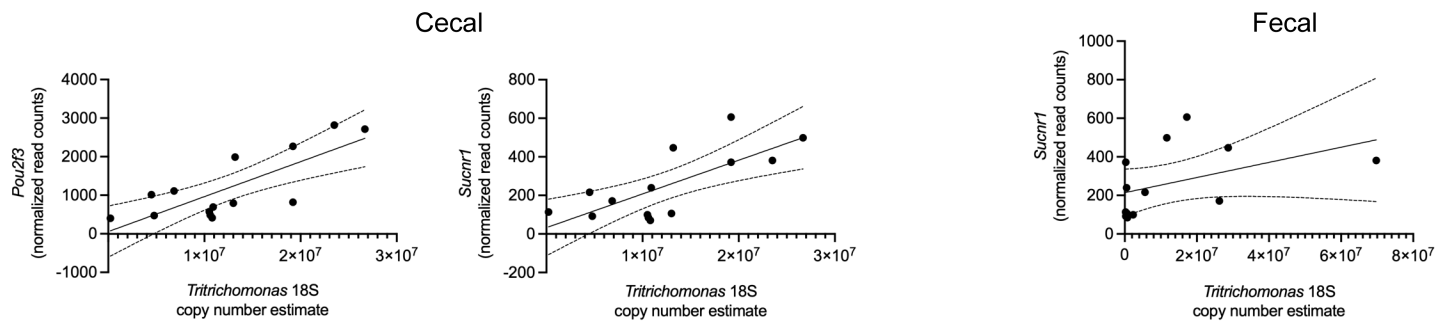

C)

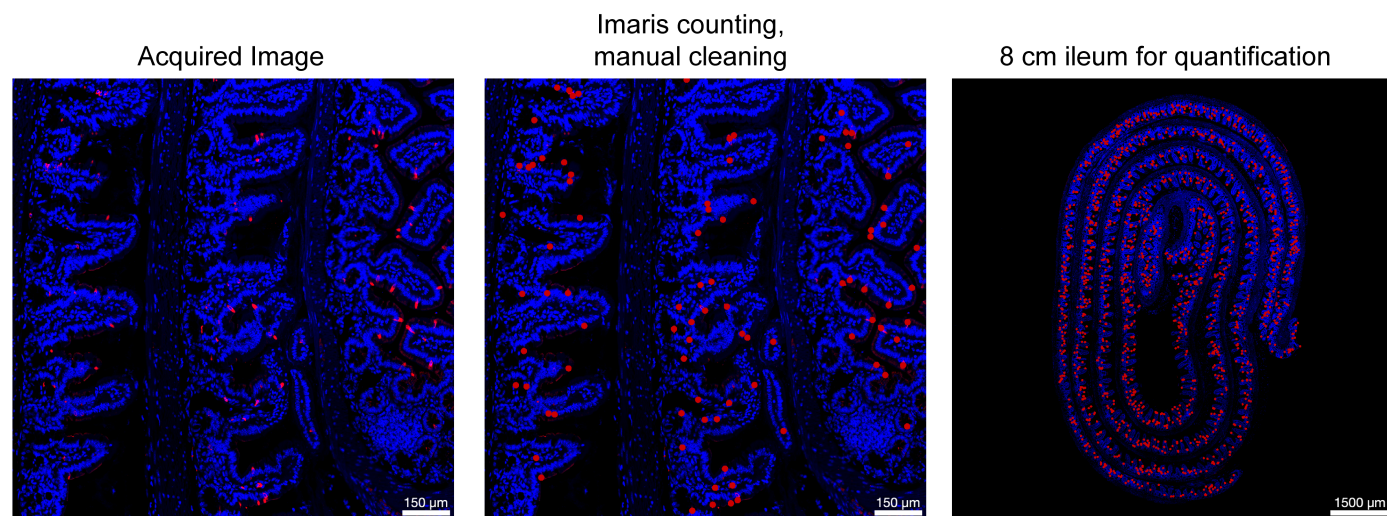

Supplement: Figure S4 — Sex-disaggregated transcriptional changes and Tritrichomonas-driven tuft cell hyperplasia in the small intestine of JAX mice infected with Tritrichomonas and E. muris. [file mbio.00802-26-s0004.pdf]

A)

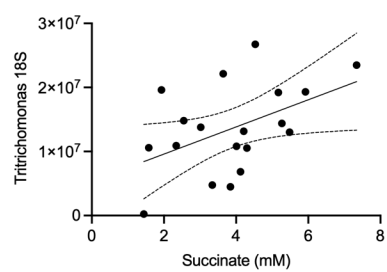

B)

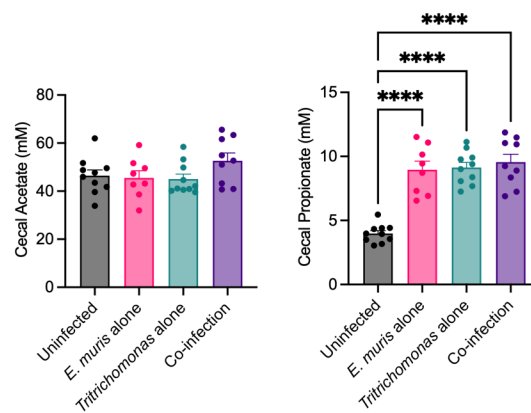

C)

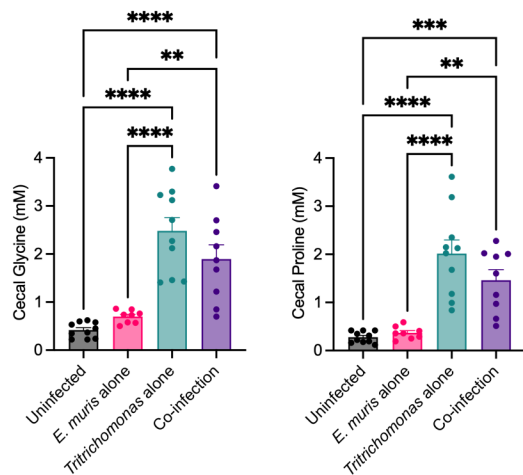

D)

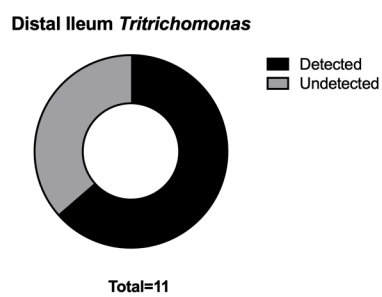

E)

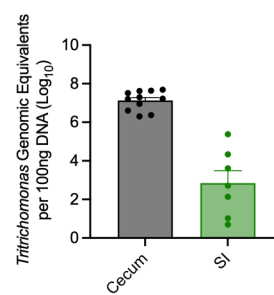

Supplement: Figure S5 — Cecal metabolite shifts induced by protist infection and variability in terminal ileum detection of Tritrichomonas spp. [file mbio.00802-26-s0005.pdf]
