# Supplementary material for: Competition between commensal protists shapes gut mucosal immunity in mice
Source: mBio. 2026 May 18;17(6):e00802-26. doi: 10.1128/mbio.00802-26 (PMC13251391; doi:10.1128/mbio.00802-26)
Supplement: Supplemental Legends — Supplemental figure and table legends. [file mbio.00802-26-s0007.docx]

**SUPPLEMENTAL FIGURE LEGENDS**

**Figure S1: *E. muris* fecal cyst-shedding and representative flow gating.**

**A-D)** Fecal cyst counts post *E. muris* infection over time. Cysts were purified via sucrose gradient centrifugation then quantified on a hemocytometer. Counts were normalized to mass of fecal starting material. **A)** Fecal cyst counts post *E. muris* infection for individual C57BL6/J mice over time.  **B)** Fecal cyst counts post *E. muris* infection of wild-type (WT) mice and *Ripk3^-/-^* mice on the C57BL/6J background. **C)** Sex segregated fecal cysts counts from **(B)**. **D)** Fecal cyst counts post *E. muris* infection for individual *Rag1^-/-^* mice over time. **E)** 18S qPCR was performed on fecal gDNA isolated daily for 3 weeks post-infection with *E. muris*. Protist burdens were determined by isolated cyst standard curve and calculated as nanograms per microliter of fecal gDNA. Representative gating strategies: **F,G)** Identification of CD45+ immune cells isolated from lamina propria. **H)** Identification of tuft cells and intraepithelial eosinophils from epithelial cell fraction. **B**,**C,E)**. Error bars represent standard error of the mean (SEM).

**Figure S2:** L**oss of intestinal tuft cell-IL-25-ILC2 circuit activity in *Tritrichomonas* spp. colonized mice infected with *E. muris*.**

**A-D)** Naturally colonized *Tritrichomonas* and protist-free mice were infected with *E. muris* as described in Figure 2A. Terminal analysis conducted at 28 dpi. **A-B)** *E. muris* shedding was quantified by qPCR from gDNA extracted from fecal pellets. **C)** Number of mice with successful *E. muris* infections. **D)** Representative flow plots for distal small intestine ILC2s and **E)** activated (Thy1-) ILC2s.

**Figure S3: Species primer analysis and cecal imaging for persistent *Tritrichomonas* spp. after *E. muris* co-infection.**

**A-E)** Mice naturally colonized with *Tritrichomonas* or protist free mice were infected with *E. muris* as described in Figure 2A. Terminal analysis conducted at 28 dpi. **A)** *Tritrichomonas* burden quantified by qPCR from gDNA extracted from fecal pellets for each mouse in the co-infection group. **B)** End point PCR utilizing species-specific primers (Gerrick et al. 2024) on purified *Tritrichomonas* spp*.* from cecal contents of in-house donor mice, indicating the presence of *T. musculus* and *T. casperi*. **C)** Representative qPCR standard curve from a synthetic 18s template (ITS) using species-specific primers for *T. musculus* (left). For comparison, the pan-species assay (right) displays the expected dilution series. *T. casperi* synthetic template failed to amplify. **D)** Individual channels of images from Figure 3D (scale bar: 50 μm). **E)** Representative images (scale bar: 100 μm) of ceca from indicated experimental groups stained with antibodies against *Tritrichomonas* (TT, pink), CD45 (green), and counterstained with DAPI (blue).

**Figure S4: Sex-disaggregated transcriptional changes and *Tritrichomonas*-driven tuft cell hyperplasia in the small intestine of JAX mice infected with *Tritrichomonas* and *E. muris*.**

**A-C)** JAX mice co-infected with *Tritrichomonas* and *E. muris* as described in Figure 4A. All analyses were conducted at 28 dpi. **A)** Heatmaps depicting gene expression of the top 500 genes by variance in the distal ileum of male (left) versus female (right) mice, row normalized z-score, unsupervised column clustering. **B)** Linear correlation of cecal *Tritrichomonas* burden based on 18S qPCR quantification versus *Pou2f3* (left, R^2^=0.6036) or *Sucnr1* (middle, R^2^=0.5404) expression by bulk RNA-sequencing of distal ileum. Fecal burden did not correlate with *Sucnr1* expression.  **C)** Imaris Spots function was used to identify tuft cells (DCLK1+) cells in distal small intestine Swiss roll sections for unbiased quantification.

**Figure S5: Cecal metabolite shifts induced by protist infection and variability in terminal ileum detection of *Tritrichomonas* spp.**

**A)** Correlation between *Tritrichomonas* spp*.* burden (genomic equivalents) and cecal content succinate concentration. **B)** Acetate and propionate concentrations in cecal content. **C)** Free amino acid concentrations of glycine and proline in cecal content. **D-E)** qPCR analysis of distal ileum content from in-house mouse lines with endemic *Tritrichomonas* colonization relative to cecal content. **D)** Detection frequency for *Tritrichomonas* in 3 cm of distal ileum. **E)** Cecal and distal Ileum (7 of 11 mice) *Tritrichomonas* burden. **B,C,E)** Error bars represent the standard error of the mean (SEM). Ordinary one-way ANOVA followed by Tukey’s multiple comparisons. ns=not significant, **p<0.005, ****p<0.0001.

**Figure S6: Colonic bulk RNA-seq suggests pan-protist changes in gene expression, but only *Tritrichomonas* infection upregulates gasdermin proteins.**

**A-E)** JAX mice co-infected with *Tritrichomonas* and *E. muris* as described in Figure 4A. All analysis conducted at 28 dpi on the proximal colon unless otherwise stated. **A)** Heatmaps depicting gene expression of the top 500 genes by variance in male (left) versus female (right) mice, row normalized by z-score, unsupervised column clustering. **B)** Heatmap depicting differential gene expression of genes related to stress/protein folding, structure/cytoskeleton and unknown function; row normalized by z-score with clustering within group. **C)** Gsdc2 and Gsdc4 expression in the distal small intestine by bulk RNA-seq. **D)** Example quantification of colon stained with an antibody against GSDMC2/GSDMC3 (Gasdermin, red). Yellow lines represent line plots across the apical epithelium used to calculate mean grey value (MGV). **E)** Representative colon images from male and female mice stained with an antibody against GSDMC2/GSDMC3 (Gasdermin, red) and counterstained with DAPI (blue). **C)** Error bars represent standard error of the mean (SEM). Ordinary one-way ANOVA followed by Tukey’s multiple comparisons. ns=not significant, *p<0.05, **p<0.005, ***p<0.0005.

**Table S1**: Differentially expressed genes from RNAseq of ileum, experimental groups vs uninfected

**Table S2**: Differentially expressed genes from RNAseq of colon, experimental groups vs uninfected

**Table S3**: Upregulated genes in colon of all infected groups relative to uninfected controls, as determined by RNAseq
