# Supplementary material for: Competition between commensal protists shapes gut mucosal immunity in mice
Source: mBio. 2026 May 18;17(6):e00802-26. doi: 10.1128/mbio.00802-26 (PMC13251391; doi:10.1128/mbio.00802-26)
Supplement: Figure S6 — Colonic bulk RNA-seq suggests pan-protist changes in gene expression, but only Tritrichomonas infection upregulates gasdermin proteins. [file mbio.00802-26-s0006.pdf]

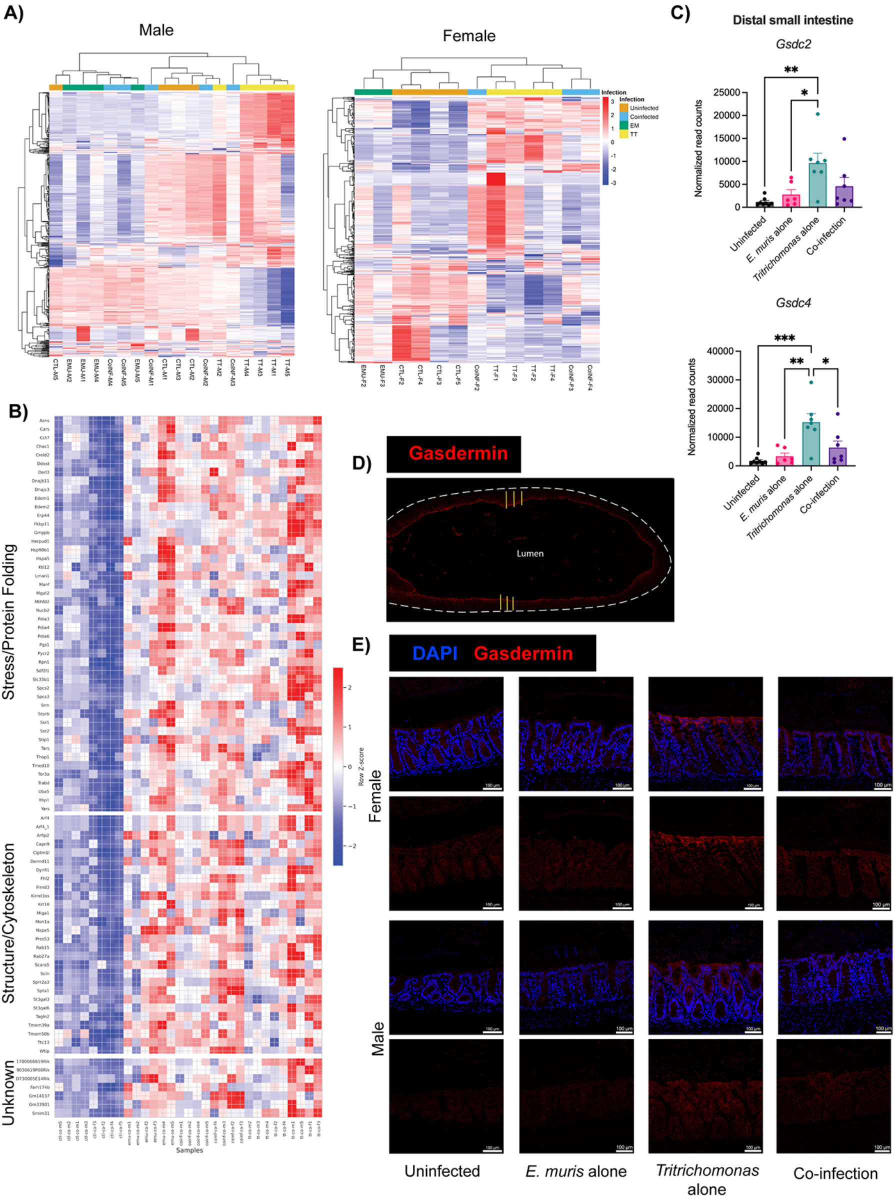
A)
Male
Female
Infection
Uninfected
Coinfected
EM
TT
B)
Stress/Protein Folding
Structure/Cytoskeleton
Unknown
Row Z-score
Samples
C)
Distal small intestine
Gsdc2
Normalized read counts
Uninfected
E. muris alone
Tritrichomonas alone
Co-infection
Gsdc4
D)
Gasdermin
Lumen
E)
DAPI Gasdermin
Female
Male
Uninfected
E. muris alone
Tritrichomonas alone
Co-infection
